# Integrin αVβ1-activated PYK2 promotes the progression of non-small-cell lung cancer via the STAT3-VGF axis

**DOI:** 10.1186/s12964-024-01639-1

**Published:** 2024-06-06

**Authors:** Zhengyan Wu, Min Jiao, Chenying Shu, Saiqun Zhang, Jiajia Wang, Jianhong Pu, Jianjie Zhu, Yuanyuan Zeng, Yehan Zhu, Zeyi Liu

**Affiliations:** 1 Department of Pulmonary and Critical Care Medicine, The First Affiliated Hospital of Soochow University, Suzhou, 215006 China; 2 Suzhou Key Laboratory for Respiratory Diseases, Suzhou, 215000 China; 3 Institute of Respiratory Diseases, Soochow University, Suzhou, 215000 China; 4 Department of Health Management Center, The First Affiliated Hospital of Soochow University, Suzhou, 215006 China; 5 Department of Pharmacy, The First Affiliated Hospital of Soochow University, Suzhou, 215006 China; 6 College of Pharmaceutical Sciences, Soochow University, Suzhou, 215123 China; 7 Department of Geriatric Medicine, The First Affiliated Hospital of Soochow University, Suzhou, 215006 China

**Keywords:** Non-small-cell lung cancer (NSCLC), PYK2, Nuclear accumulation of *p*-STAT3(Tyr705), VGF, Integrin αVβ1

## Abstract

**Background:**

Non-small-cell lung cancer (NSCLC) accounts for 80–85% of all lung cancer and is the leading cause of cancer-related deaths globally. Although various treatment strategies have been introduced, the 5-year survival rate of patients with NSCLC is only 20–30%. Thus, it remains necessary to study the pathogenesis of NSCLC and develop new therapeutic drugs. Notably, PYK2 has been implicated in the progression of many tumors, including NSCLC, but its detailed mechanism remains unclear. In this study, we aimed to elucidate the mechanisms through which PYK2 promotes NSCLC progression.

**Methods:**

The mRNA and protein levels of various molecules were measured using qRT-PCR, western blot (WB), and immunohistochemistry (IHC), respectively. We established stable PYK2 knockdown and overexpression cell lines, and CCK-8, EdU, and clonogenic assays; wound healing, transwell migration, and Matrigel invasion assays; and flow cytometry were employed to assess the phenotypes of tumor cells. Protein interactions were evaluated with co-immunoprecipitation (co-IP), immunofluorescence (IF)-based colocalization, and nucleocytoplasmic separation assays. RNA sequencing was performed to explore the transcriptional regulation mediated by PYK2. Secreted VGF levels were examined using ELISA. Dual-luciferase reporter system was used to detect transcriptional regulation site. PF4618433 (PYK2 inhibitor) and Stattic (STAT3 inhibitor) were used for rescue experiments. A public database was mined to analyze the effect of these molecules on NSCLC prognosis. To investigate the role of PYK2 in vivo, mouse xenograft models of lung carcinoma were established and examined.

**Results:**

The protein level of PYK2 was higher in human NSCLC tumors than in the adjacent normal tissue, and higher PYK2 expression was associated with poorer prognosis. PYK2 knockdown inhibited the proliferation and motility of tumor cells and caused G1-S arrest and cyclinD1 downregulation in A549 and H460 cells. Meanwhile, PYK2 overexpression had the opposite effect in H1299 cells. The siRNA-induced inhibition of integrins alpha V and beta 1 led to the downregulation of *p*-PYK2(Tyr402). Activated PYK2 could bind to STAT3 and enhance its phosphorylation at Tyr705, regulating the nuclear accumulation of *p*-STAT3(Tyr705). This further promoted the expression of VGF, as confirmed by RNA sequencing in a PYK2-overexpressing H1299 cell line and validated by rescue experiments. Two sites in promoter region of VGF gene were confirmed as binding sites of STAT3 by Dual-luciferase assay. Data from the TGCA database showed that VGF was related to the poor prognosis of NSCLC. IHC revealed higher *p*-PYK2(Tyr402) and VGF expression in lung tumors than in adjacent normal tissues. Moreover, both proteins showed higher levels in advanced TNM stages than earlier ones. A positive linear correlation existed between the IHC score of *p*-PYK2(Tyr402) and VGF. Knockdown of VGF inhibited tumor progression and reversed the tumor promoting effect of PYK2 overexpression in NSCLC cells. Finally, the mouse model exhibited enhanced tumor growth when PYK2 was overexpressed, while the inhibitors PF4618433 and Stattic could attenuate this effect.

**Conclusions:**

The Integrin αVβ1-PYK2-STAT3-VGF axis promotes NSCLC development, and the PYK2 inhibitor PF4618433 and STAT3 inhibitor Stattic can reverse the pro-tumorigenic effect of high PYK2 expression in mouse models. Our findings provide insights into NSCLC progression and could guide potential therapeutic strategies against NSCLC with high PYK2 expression levels.

**Supplementary Information:**

The online version contains supplementary material available at 10.1186/s12964-024-01639-1.

## Introduction

In 2019, there were 2.26 million new cases of lung cancer and 2.04 million deaths due to lung cancer globally, making lung cancer the most common type of cancer and the leading cause of cancer-related mortality in the world [1]. According to the most recent estimates, lung cancer has the second-highest incidence among all cancers in the United States. It has been responsible for the highest number of cancer-related deaths in the country (21%) in 2023 [2]. Notably, lung cancer also has the highest incidence rate among all malignancies in China [3].

Non-small-cell lung cancer (NSCLC), an aggressive malignancy, accounts for approximately 80–85% of all lung cancers and includes squamous cell carcinoma, adenocarcinoma, large cell carcinoma, lung sarcoma, and other types of lung cancer. So far, several therapeutic strategies have been employed to treat NSCLC, including chemotherapy, radiotherapy, immunotherapy, and gene-targeted therapy. However, the efficacy of these therapies has been limited in the later stages of treatment. Although the age-standardized 5-year relative survival of NSCLS has increased over the last few years, according to the latest statistics, it remains at only 26.4% [4]. Hence, further research on the mechanisms of NSCLC is urgently warranted to develop improved treatment strategies.

Proline-rich tyrosine kinase 2 (PYK2), encoded by the protein tyrosine kinase two beta (*PTK2B*) gene, was first identified as a non-receptor tyrosine kinase belonging to the focal adhesion kinase (FAK) family in 1995 [5]. PYK2 is expressed in the cytoplasm and nucleus [6] and is composed of an N-terminal protein 4.1, ezrin, radixin, moesin (FERM) domain, a short FERM–kinase linker that contains the proline-rich 1 (PR1) sequence, a central tyrosine kinase domain, a longer kinase–focal adhesion targeting (FAT) linker containing the proline-rich 2–3 (PR2-3) sequence, and a C-terminal FAT domain. PYK2 has many binding sites for signal transduction and can be activated by Ca^2+^ influx or by binding to other molecules such as growth factors, chemokines/cytokines and their receptors, and integrins [6]. PYK2 upregulation has been identified in various hematologic neoplasms [7, 8]and solid tumors and is associated with epithelial–mesenchymal transition (EMT), cell proliferation, ROS activity, metabolism, anchorage-independent growth, and stemness in tumor cells. Moreover, high levels of PYK2 are associated with increased tumor angiogenesis and drug resistance, as well as with a poor prognosis [9–21]. Several studies have shown that PYK2 is involved in NSCLC progression and is associated with poor outcomes in patients with NSCLC [11, 12, 22–24]. Nevertheless, the molecular mechanisms underlying the role of PYK2 in NSCLC remain unclear.

In this study, we aimed to elucidate the role of PYK2 in NSCLC progression and the mechanisms underlying its action. We found that PYK2 and its activated form, *p*-PYK2 (phosphorylated at Tyr402), are upregulated in NSCLC, and high PYK2 levels are related to a poor prognosis. Then, we explored the probable molecular pathways involved in the effects of PYK2. To this end, we investigated potential upstream activators. We tested the impact of the PYK2 inhibitor PF4618433 and the downstream signal transducer and activator of transcription 3 (STAT3) inhibitor Stattic on tumor cell growth in vitro and in vivo. Our findings provide insights into the progression of NSCLC and reveal potential therapeutic strategies against NSCLC with high PYK2 expression levels.

## Materials and methods

### Tissue samples and serum

Samples of NSCLC tumor tissue, adjacent noncancerous lung tissue, and serum from patients and healthy controls were collected from the First Affiliated Hospital of Soochow University (Ethics number 82073213). Informed consent was obtained from all individuals before the examination. Patients were diagnosed with NSCLC based on tumor histology and pathology per the revised International System for Staging Lung Cancer. None of the patients had undergone chemotherapy or radiotherapy before tissue sampling. The tissue samples were stored at -80 °C in a cryogenic freezer. The First Affiliated Hospital of Soochow University Academic Advisory Board approved this study. Details are provided in the supplemental materials.

### Cell lines and culture

We purchased the NSCLC cell lines A549, H1299, H1650, HCC827, H226, and H460 from the Cell Bank of the Chinese Academy of Sciences (Shanghai, China). All tumor cells were cultured at 37 °C under 5% CO_2_ in F12K or RPMI-1640 medium supplemented with 10% fetal bovine serum (Gibco, Carlsbad, CA) and 1% penicillin-streptomycin (Invitrogen, Carlsbad, CA, USA).

### **RNA extraction and quantitative real-time polymerase chain reaction (qRT-PCR)**

Total RNA was extracted from cells using the TRIzol reagent (15,596,026; Thermo Fisher Scientific) according to the manufacturer’s instructions. The RNA was reverse transcribed into cDNA using M-MLV Reverse Transcriptase (TaKaRa, Osaka, Japan). PCR was performed using SYBR® Premix Ex Taq™ (TaKaRa, Osaka, Japan) and the ABI Step One Plus Real-Time PCR system (Applied Biosystems, Foster City, CA, USA). Primers are listed in supplemental Table 1. The relative expression of target genes was calculated using the 2^−ΔΔCt^ method, with β-actin as the internal control.

### Establishment of stable PYK2 knockdown and PYK2 overexpression cell lines

We purchased PYK2 knockdown, overexpression, and control plasmids from GeneChem Corporation (Shanghai, China). HEK293T cells were co-transfected with a lentivirus vector and packaging system and cultured for approximately 48-72 h before collecting the culture medium containing the virus particles. Cells were selected with 2 µg/mL puromycin (Sigma-Aldrich, St Louis, MO, USA) to establish stable cell lines for subsequent experiments.

### Western blot (WB)

WB was performed according to routine protocols [25]. The antibodies used are listed in supplemental Table 2.

### Cell proliferation analysis and EdU incorporation assay

The Cell Counting Kit-8 (CCK-8) assay (Beyotime, Shanghai, China) was used to measure cell proliferation. Cells were seeded in 96-well plates (3 × 10^3^) cells per well and cultured for 24, 48, and 72 h under standard culture conditions. The viability of the cells was measured three times based on the manufacturer’s instructions.

Cell proliferation was determined using an EdU (5-ethynyl-2-deoxyuridine) assay (Ribobio). Stable A549, H460, and H1299 cells were plated in 96-well plates and exposed to EdU for two hours. After 48 h, the cells were fixed in 4% formaldehyde for 30 min at 20℃. Following neutralization with glycine and washing, cells were treated with 0.5% Triton X-100 for 30 min and then incubated with the Apollo® reaction cocktail for 30 min. Nuclei were stained with 100 µL Hoechst 33,342 (5 µg/mL) for 5 min. Using Image J software, EdU-positive cells were counted under a fluorescent microscope (Olympus).

### Colony formation assay

A colony formation assay was performed to confirm the malignant transformation. To this end, 3 × 10^3^ cells were seeded per well and cultured in 5% CO_2_ at 37 °C. ImageJ was used to count colonies after 7-14 days.

### Migration and invasion assays

Migration and invasion assays were performed using Corning Transwell inserts (8.0 μm pore size) (Corning, New York, NY, USA). Transwell inserts were filled with 800 µL of F12K or RPMI-1640 medium containing 10% FBS for migration assays. Then, 4 × 10^4^ trypsinized stable cells were seeded into medium containing 1% FBS and incubated in a humidified incubator at 37 °C for 24 h (H460 cell line, 48 h). Cells that migrated toward the lower surface of the insert were fixed for 30 min with 100% methanol, dried for 10 min, stained with 0.1% crystal violet for 30 min, and washed with 1× phosphate-buffered saline (PBS) thrice. The inserts were coated with Matrigel (BD Science, Sparks, MD, USA) diluted in serum-free medium in the invasion assay. The cells were then incubated at 37 °C for two hours, and the remaining procedures were carried out as described for the migration assay. A microscope (CKX41, Olympus) was used to acquire images of the cells. Cells were counted in at least three microscopic fields (magnification, ×100). Each test was repeated thrice.

### Cell cycle analysis

Cells were cultured in 6-well plates for 72 h based on the instructions provided by the Cell Cycle Analysis Kit (Beyotime, Shanghai, China). The cells were collected and washed in cold PBS before being fixed in 70% ethanol for 24 h at 4 °C. The cells were again washed in cold PBS and stained with propidium iodide (PI) and an RNaseA mixture. Following this, cells were incubated at 37 °C for 30 min before analysis using a fluorescent-activated cell sorting (FACS) system (Beckman Coulter, Brea, CA).

### Transfection

The stable cell lines A549 and H1299 were seeded in 6-well plates. After the cells reached 40-60% confluence, they were transfected using the jet PRIME reagent (Invitrogen) according to the manufacturer’s instructions. For further experiments, cells were collected 48-72 h after transfection. GenePharmacompany (Suzhou, China) provided the ITGA3, ITGA5, ITGAV, ITGA6, and ITGB1 siRNAs and the corresponding controls. A list of the target siRNA sequences is provided in the supplemental material.

### Co-immunoprecipitation (co-IP) assay

A 100-mm plate of 95-100% confluent NSCLC cells was used for endogenous co-IP. HEK293T cells seeded in 6-well plates were transfected with two plasmids (PYK2-FLAG plasmid obtained from GeneChem, Shanghai, and STAT3-MYC plasmid generously gifted by Dr Zeng Yuanyuan) for 48 h to express the proteins for exogenous co-IP. After a double wash with cold PBS, the cells were scraped off. The cells were then lysed for 30 min at four °C in 1 mL of modified IP cracking solution (Beyotime, Shanghai, China) containing a cocktail of protease and phosphatase inhibitors (Sigma-Aldrich, St. Louis, MO, USA). The lysate was centrifuged at 12,000 *g* and 4 °C for 15 min. Subsequently, the supernatants were transferred to fresh Eppendorf tubes and incubated overnight with 1-2 µg of IgG or antibodies against the target protein. The overnight incubation of clear lysates at four °C, with rotation, was followed by adding ten µL of protein A/G beads. The beads were washed with IP cracking solution thrice and boiled for 5 min at 100 °C in 2× SDS protein-loading buffer. Western blot analysis was performed after loading ten µL of the samples on SDS-PAGE gels.

### Immunofluorescence (IF) staining

First, 4% paraformaldehyde was used to fix cells for 15 min at room temperature. Then, the cells were rinsed thrice with PBS and incubated with 5% BSA for one hour at room temperature before blocking. Finally, the cells were incubated with antibodies against PYK2, Integrin alpha V, Integrin beta 1, *p*-PYK2(Tyr 402), and STAT3 overnight at 4 °C. Detailed antibody information is provided in the supplemental material. Alexa Fluor 647- and FITC- tagged secondary antibodies (1:500, Beyotime Biotechnology) were used to obtain readout signals. Cells were incubated in DAPI (Life Technologies) for counterstaining for 2 min. Fluorescent markers were finally imaged using the Leica SP8 confocal microscope.

### Nuclear and cytoplasmic extraction

The ProteinExt™ Mammalian Nuclear and Cytoplasmic Protein Extraction Kit (TRANS, China) was used to separate nuclear and cytoplasmic proteins. Confluent H1299 cells transfected with the empty vector and PYK2-overexpression vector were harvested. Then, the standard procedure was performed, as described in the manufacturer’s instructions.

### mRNA sequencing

Confluent 100-mm wells of H1299 vector and H1299 PYK2-overexpressing stable cells were lysed with 1 mL of RNAiso Plus (TaKaRa, Osaka, Japan). RNA sequencing was performed on an Illumina HiSeq XTen sequencer (NovelBio Bio-Pharm Technology Co., Ltd., Shanghai, China). Evaluations between the two groups were conducted in a 3:3 ratio.

### ELISA

According to the manufacturer’s instructions, the secreted VGF nerve growth factor inducible (VGF) levels were measured using a VGF-ELISA Kit (Finetest, Wuhan, China). The serum concentration of VGF was detected in 19 NSCLC patients and 10 healthy controls (Ethics number 82073213). The clinical information is provided in the supplemental material. A549, H460, and H1299 stable cell lines were seeded in 6-well plates, and cell culture supernatants were collected for VGF ELISA as soon as the cell density reached 95-100%.

### Immunohistochemistry (IHC) assay

IHC was performed as described previously [25], and the antibodies and dilutions used are listed in the supplemental materials. Tissue microarrays (ZL-Lug1201) purchased from Weiaobio (Shanghai, China) were used to detect *p*-PYK2(Tyr402) and VGF protein levels in paired lung cancer tissues and adjacent normal tissues (Ethics number LLS M-15-01). Data from three pairs of tissues were excluded, and finally, 57 paired tissues from NSCLC patients were analyzed.

### Luciferase reporter assay

Luciferase reporter assay was carried out as described previously [26]. Primers used were listed in Table S6.

### Animal experiments

Athymic nude BALB/c mice (3-4 weeks old and weighing 16-20 g) were obtained from the Experimental Animal Center of Soochow University and bred in a pathogen-free environment. All animal experiments complied with the Guidelines for the Care and Use of Experimental Animals Center at Soochow University. Two primary in vivo experiments were conducted (Ethics number 202303A0334).

In the first experiment, A549 negative control and A549 PYK2-knockdown stable cells (3 × 10^6^ cells per mouse) were transplanted subcutaneously into the armpit regions of the nude mice. The width and length of the xenografts were measured throughout the experimental period.

In the second experiment, the A549 vector and A549 PYK2-overexpressing stable cells were suspended in cell culture medium without fetal bovine serum and inoculated subcutaneously with Matrigel into the flanks of nude mice. Five mice were in the control group and 15 in the PYK2 overexpression group. When the tumors neared a size of 100 mm^3^, 10 of the mice from the PYK2 overexpression group were randomly divided into groups of 5 each. The first group received intraperitoneal injections of the PYK2 inhibitor PF4618433 (HY-18,312, MCE, USA; 2 mg/kg), and the second group received the STAT3 inhibitor Stattic (HY-13,818, MCE, USA; 2 mg/kg) [27] once every two days until the end of the experimental period. We also measured the body weight of mice every two days. To determine tumor volume (V), we measured tumor length (L) and width (W) using a Vernier caliper and used the following formula: V = (L × W^2^) × 0.5.

The mice were sacrificed when the most enormous tumor volume reached nearly 1000 mm^3^. Xenograft tumors were harvested for hematoxylin and eosin (H&E) staining, IHC, and WB.

### Statistical analysis

Statistical analysis was performed using R (Version 4.0.2), Gene Set Enrichment Analysis (GSEA), and GraphPad Prism (8.0). We used R to analyze the effect of PYK2 and VGF expression on the overall survival of NSCLC patients using TCGA data. Experimental data were presented as the mean ± standard deviation. Based on data distribution and variance homogeneity, data were compared between groups using the t-test, Welch’s t-test, or Mann-Whitney U-test as appropriate. The paired t-test and Wilcoxon paired t-test were used to compare and analyze the difference in parametric and non-parametric variables between tumors and adjacent normal tissues. Pearson or Spearman correlation analysis was performed to evaluate correlations between quantitative variables, depending on whether the data showed normal distribution. Data comparisons among three or more groups were performed using a one-way or two-way ANOVA, followed by Benjamini’s post hoc test. We considered two-tailed P values of less than 0.05 statistically significant. * *P* < 0.05, ** *P* < 0.01, *** *P* < 0.001, **** *P* < 0.0001.

## Results

**PYK2 and ***p*-PYK2(Tyr402) **are highly expressed in NSCLC tissues and ***PTK2B* mRNA **is associated with a poor prognosis**.

**Fig. 1:**
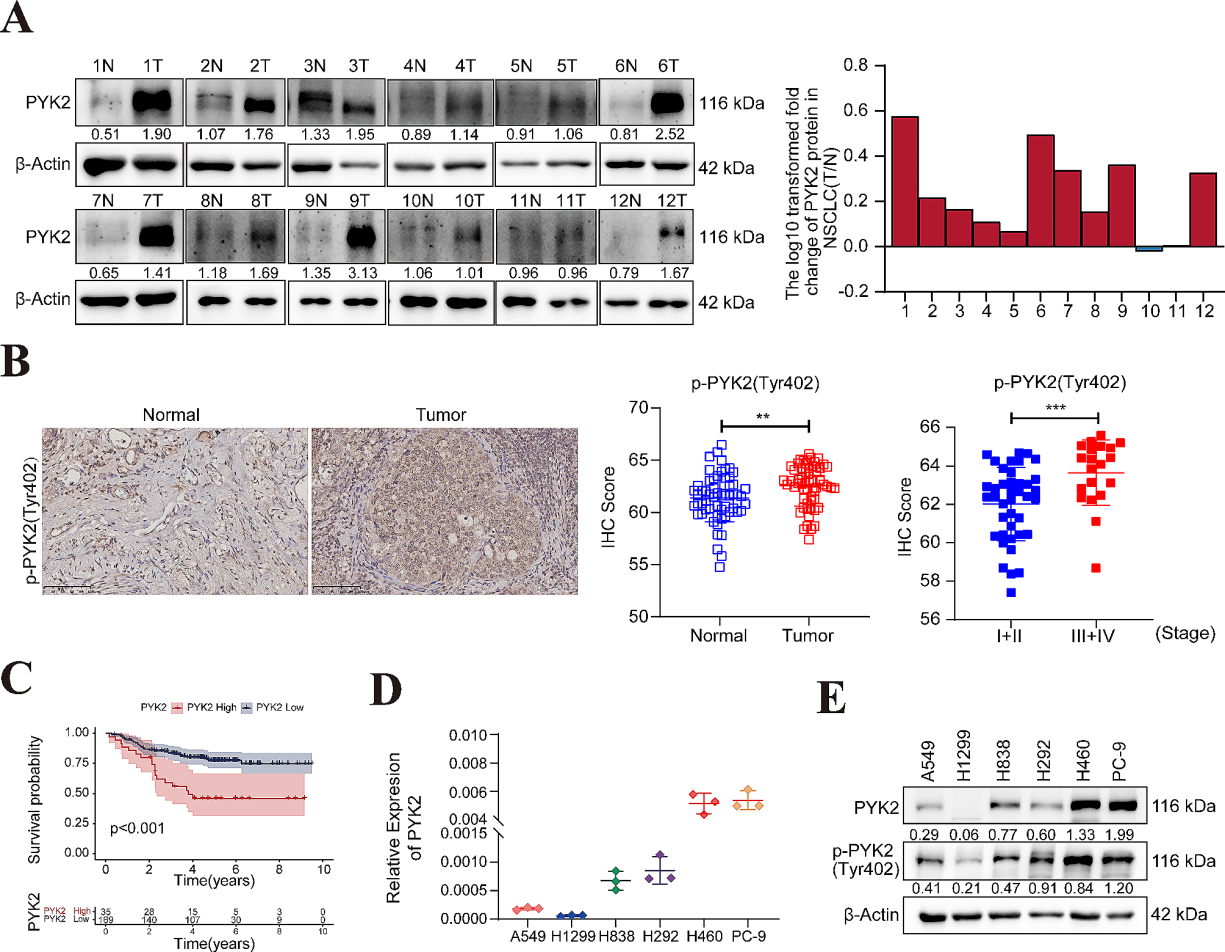
PYK2 and *p*-PYK2(Tyr402) are highly expressed in NSCLC tissues and *PTK2B* mRNA is associated with a poor prognosis. **A** PYK2 protein expression level of NSCLC tissues is higher than paired normal lung tissues. **B** IHC indicate the higher expression of *p*-PYK2(Tyr402) in NSCLC tumor than adjacent nontumor tissue, and higher expression in later TNM stage than earlier TNM stage. Scale bar, 100 μm. **C***PTK2B* expression level is negatively related with the overall survival of NSCLC patients. The expression and prognostic data were downloaded from the TCGA database. **D-E***PTK2B* mRNA and PYK2, *p*-PYK2(Tyr402) protein levels in NSCLC cell lines. * *P* < 0.05; ** *P* < 0.01; *** *P* < 0.001; **** *P* < 0.0001

The protein level of PYK2 and its major activated form *p*-PYK2(Tyr402) were higher in tumor tissues than in paired adjacent normal tissues detected by WB and IHC(Fig. 1A-B). the IHC scores of *p*-PYK2(Tyr402) were also significantly higher in advanced TNM stages than in early TNM stages. We used TCGA data (https://portal.gdc.cancer.gov/) to analyze *PTK2B* mRNA expression and its association with prognosis. Kaplan-Meier curves revealed a correlation between higher *PTK2B* mRNA expression and poorer overall survival among 204 patients with NSCLC (Fig. 1C). Next, a variety of NSCLC cell lines were tested for PTK2B mRNA and PYK2, *p*-PYK2(Tyr402) protein levels (Fig. 1D-E). We selected A549 cells (with medium PYK2 expression), H460 cells (with higher PYK2 expression), and H1299 cells (with lower PYK2 expression) for follow-up experiments.

**Altered PYK2**, *p*-PYK2(Tyr402) **expression affects NSCLC cell proliferation**, **migration, and invasion** in vitro.

**Fig. 2:**
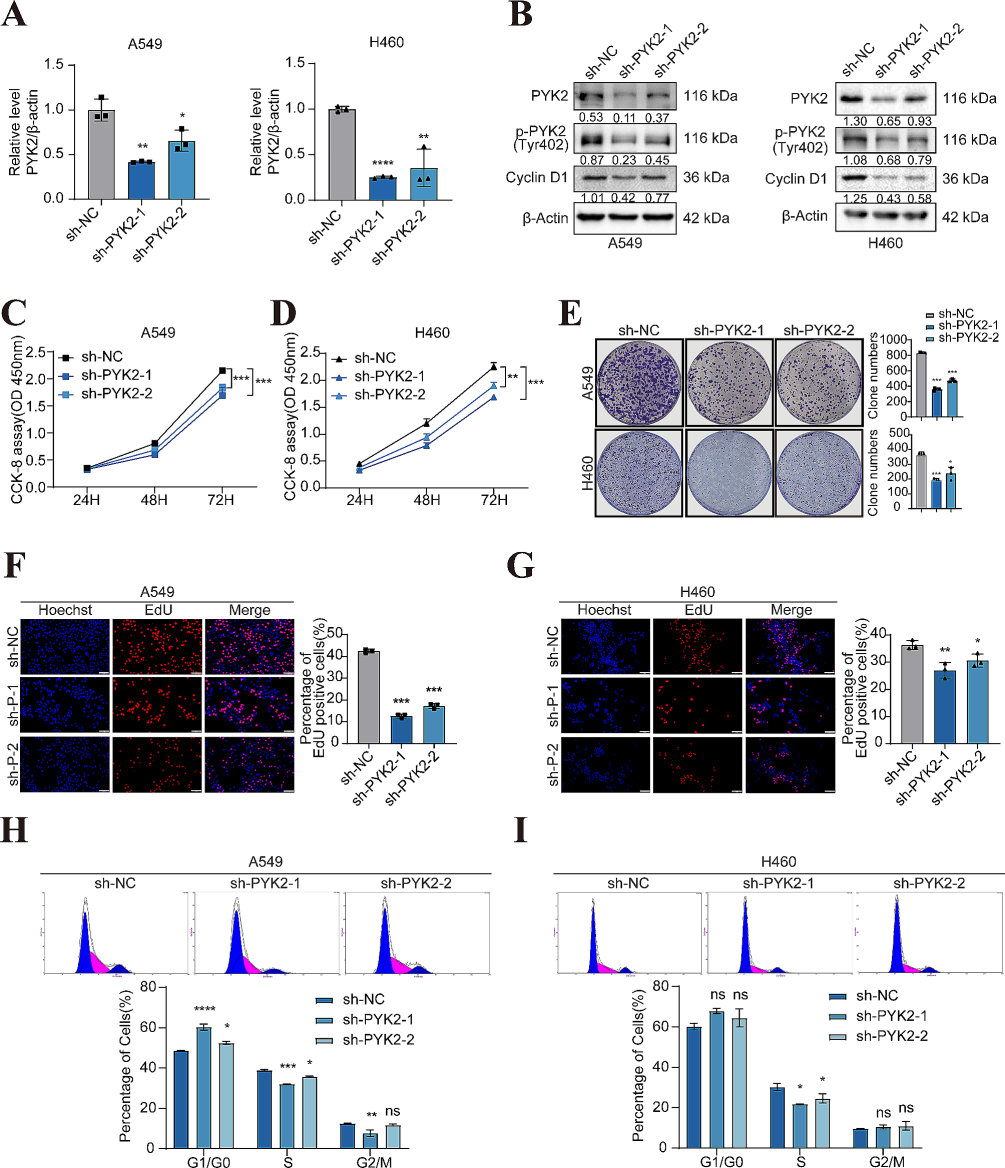
Inhibition of NSCLC cell proliferation and cell cycle G1-S arrest by knockdown of PYK2. **A-B** PYK2 mRNA and protein levels were knockdown in A549 and H460 sh-PYK2 cells. **C-D** CCK-8 assay was used to detect cell viability in A549 and H460 cells; cell viability was determined at 24, 48, and 72 h. PYK2-knockdown inhibited growth of A549 and H460 cells. **E** PYK2-knockdown inhibited the clonogenic ability of A549 and H460 cells. **F-G** EdU further confirmed that PYK2 knockdown inhibited cell proliferation. Scale bar, 200 μm. **H-I** Knockdown of PYK2 caused cell cycle G1-S arrest of A549 and H460 cells. * *P* < 0.05; ** *P* < 0.01; *** *P* < 0.001; **** *P* < 0.0001

**Fig. 3:**
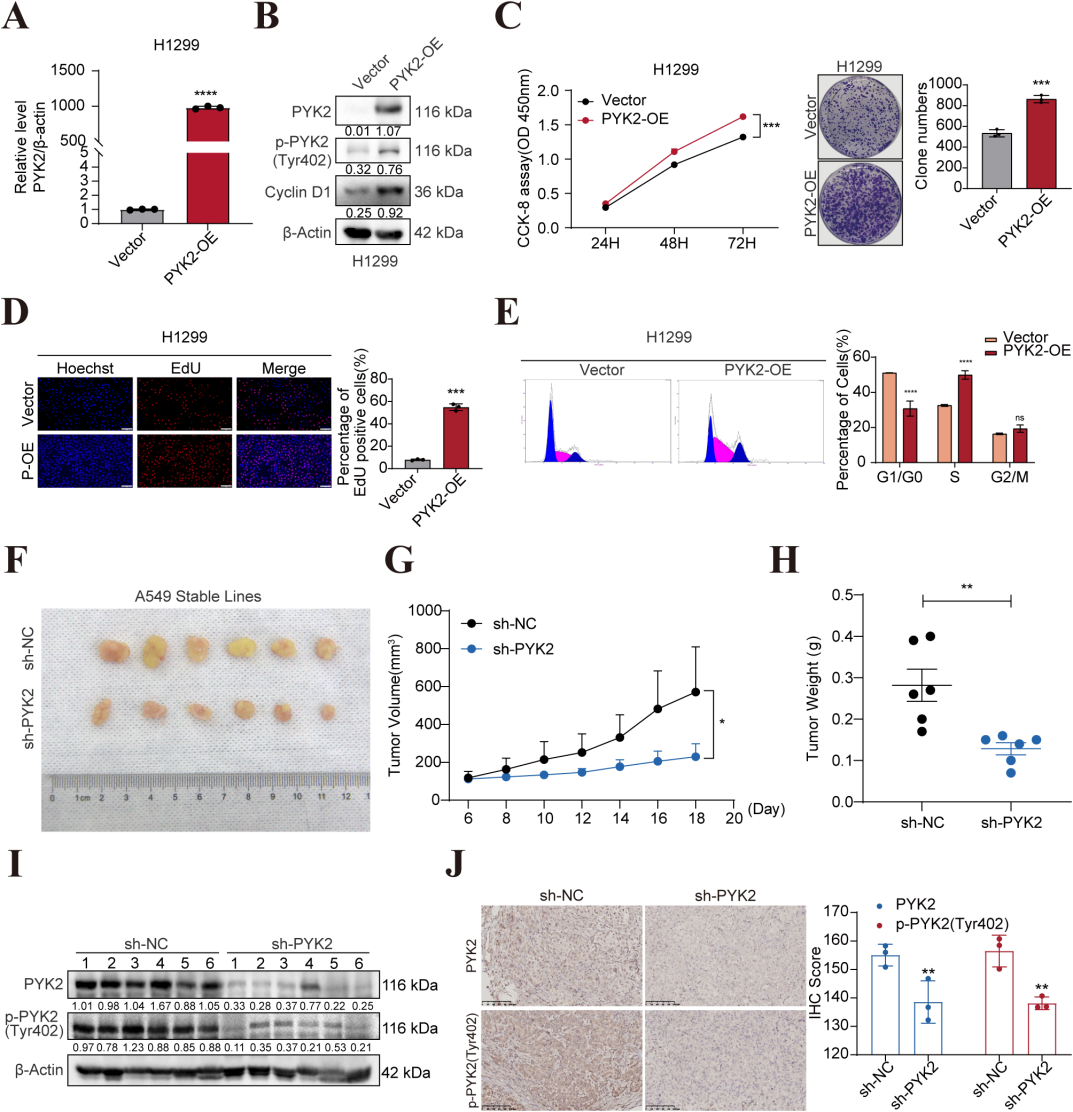
PYK2-overexpression promoted the proliferation of H1299 cells, knockdown of PYK2 attenuates tumor growth in a murine xenograft model. **A-B** PYK2 mRNA and protein levels increased in H1299 PYK2 overexpressed cells. **C** CCK-8 assay to detect cell viability in H1299 cells; PYK2-overexpression promoted the clonogenic ability of H1299 cells. **D** EdU further confirmed that PYK2 overexpression enhanced cell proliferation. Scale bar, 200 μm. **E** Overexpression of PYK2 caused cell cycle G1 stage transfer to S stage in H1299 cells. **F** PYK2-knockdown in A549 cells xenografts in nude mice (*n* = 6) at the experimental endpoint. Tumors were dissected and photographed as shown. **G** Tumor growth curves in mice (*n* = 6 in each group). **H** Each tumor formed was weighted. **I-J** WB and IHC detected *p*-PYK2 expression and the total PYK2 expression in tumor cell. * *P* < 0.05; ** *P* < 0.01; *** *P* < 0.001; **** *P* < 0.0001

To further investigate the function of PYK2 in NSCLC cells, stable PYK2 knockdown was achieved in A549 and H460 cell lines (Fig. 2A-B), and H1299 cells were chosen to construct a stable PYK2 overexpression line (Fig. 3A-B). Changes in *p*-PYK2(Tyr402) and cyclinD1 levels were consistent with the changes in PYK2 protein levels. CCK-8, EdU, and clonogenic assays revealed that, down-regulation of PYK2 inhibited the ability of cell proliferation (Fig. 2C-G) and up-regulation of PYK2 promotes NSCLC cell proliferation (Fig. 3C-D). The transwell assay also demonstrated that down-regulation of PYK2 inhibited the ability of cell migration and invasion, up-regulation of PYK2 enhanced cell migration and invasion in vitro (Fig. S1A-C). Moreover, the number of cells in the S phase and the G0/G1 phase changed after PYK2 expression was perturbed, suggesting that over expression of PYK2 may promote the G1-S phase transition during the cell cycle, on the contrary, knockdown of PYK2 caused cell cycle G1-S arrest (Figs. 2H-I and 3E).

### Knockdown of PYK2 attenuates tumor growth in a murine xenograft model

To further evaluate the effects of PYK2 knockdown in vivo, xenograft models of athymic BALB/c mice were established. According to the findings, tumors generated from cells with PYK2 knockdown were much smaller in size than those generated from control cells, and PYK2 knockdown reduced the growth rate of xenografts as well as the final tumor weight (Fig. 3F-H). Xenograft tumor tissues were also analyzed. WB and IHC analysis confirmed that the levels of PYK2 and *p*-PYK2(Tyr402) were decreased in the knockdown group (Fig. 3I-J). Thus, the findings of in vivo experiments were consistent with those obtained in vitro.

### Integrin αVβ1 signaling regulates the phosphorylation of PYK2 at tyrosine 402

**Fig. 4:**
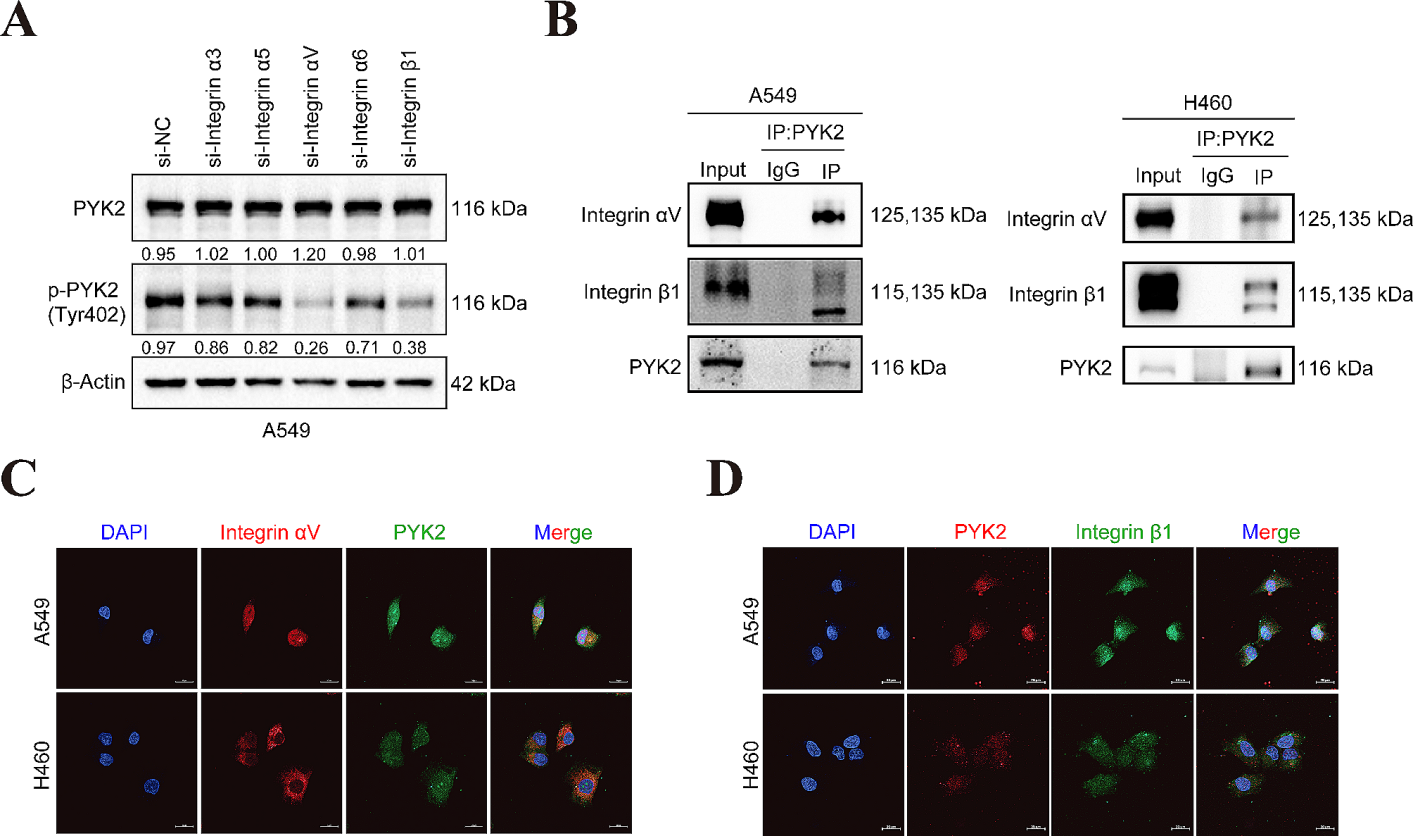
Knockdown of Integrin αVβ1 could inhibit the tyrosine 402 site phosphorylation of PYK2. **A** Transient transfection of siRNAs of Integrin α3, α5, αV, α6, and β1 to screen which one could affect the activation of PYK2 and WB to detect *p*-PYK2(tyr402) in knockdown of each Integrin in A549 cells; **B** CO-IP demonstrated the interaction of PYK2 and Integrin αV, β1.**C-D** Colocalization of PYK2 and Integrin αV, β1 (PYK2, SANTACRUZ, sc-393,181, Mouse for A and PYK2, abcam, ab32571, Rabbit for B) by IF. Scale bar, 20 μm

As PYK2 is a non-receptor tyrosine kinase, its kinase is activated by tyrosine phosphorylation at the 402 site [28]. The full-length PYK2 can form oligomeric complexes within cells, and the complexes are positively correlated with 402 tyrosine phosphorylation [29], also our Figs. 2 and 3 results confirmed that when PYK2 was knockdown or overexpressed, the protein level of *p*-PYK2(Tyr402) was always consistent of PYK2 and was correlated with NSCLC progression. In addition to the increase in total PYK2 expression, how can its kinase activity be activated ? In previous studies of our laboratory, we found that integrin α3/α6 and αV [30], β1 [31, 32] play a promoting role in the progression of non-small cell lung cancer and could activate FAK, another member of PYK2 subfamily. So we assumed that integrins may activate PYK2. To identify which integrin may regulate the expression of PYK2 in lung cancer cells, integrins α3, α5, αV, α6, and β1 were knocked down in A549 cells (Figure S2 A-E), and the effects on PYK2 activation were examined. The results showed that integrin αVβ1 was responsible for inducing the most common activated form of PYK2: *p*-PYK2(tyr402) (Fig. 4A). IF staining and co-IP assays confirmed the colocalization and interaction of Integrin αVβ1 with PYK2 in A549 and H460 cells (Fig. 4B-D). We also found the negative correlation of integrin αV and β1 mRNA level on the prognosis of NSCLC patients based on the public TCGA database, which was shown in Figure S2F-G.

*p*-PYK2(Tyr402) **interacts with STAT3 and can regulate the phosphorylation of STAT3 at tyrosine 705 and the nuclear accumulation of ***p*-STAT3(Tyr705).

**Fig. 5:**
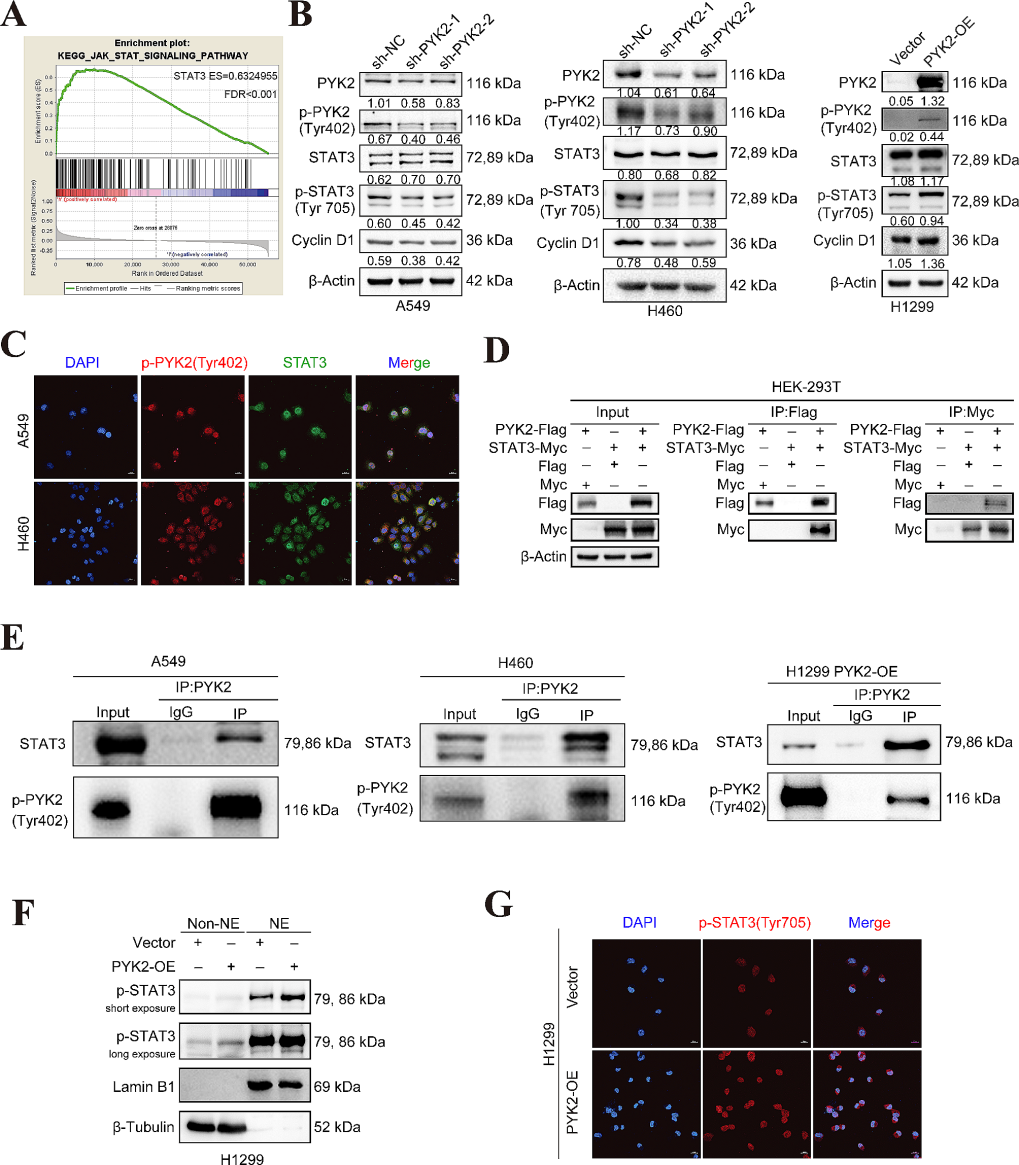
*p*-PYK2(Tyr402) interacts with STAT3 and can regulate the tyrosine 705 phosphorylation of STAT3 and *p*-STAT3(Tyr705) nuclear accumulation. **A** GSEA analysis showed a positive relation between PYK2 and the JAK-STAT signaling pathway. **B** Level of total STAT3 and *p*-STAT3(Tyr705) in A549, H460 stable PYK2 knockdown stable cell lines and H1299 stable PYK2 overexpressed cell line. **C ***p*-PYK2(Tyr402) and STAT3 colocalized in A549 and H460 cells. **D** Coimmunoprecipitation assay of exogenously expressed PYK2 and STAT3 plasmid in HEK293T cell demonstrated the interaction of these two proteins. **E ***p*-PYK2(Tyr402) and STAT3 could be directly bound to each other. **F-G** Nucleocytoplasmic separation and immunofluorescence experiments showed more *p*-STAT3(Tyr705) nuclear accumulation in PYK2 overexpressed compared to the vector

It has been reported that PYK2 is upstream of STAT3 in hematological malignancies [8]. However, their relationship to lung cancer is still unknown. We performed GSEA using TCGA datasets (https://portal.gdc.cancer.gov/) and found that the KEGG_JAK_STAT signaling pathway was enriched (NES = 2.6638682, FDR < 0.001) (Fig. 5A). In the pathway, the enrichment score (ES) of STAT3 was 0.6324955. Hence, we tested total STAT3 and *p*-STAT3(Tyr705) levels in the A549 and H460 stable PYK2 knockdown cells and the H1299 stable PYK2 overexpression cell. WB showed that PYK2/*p*-PYK2(tyr402) positively regulated *p*-STAT3(tyr705) expression but did not significantly affect total STAT3 levels (Fig. 5B). We further investigated the correlation between *p*-PYK2(Tyr402) and STAT3 expression using co-IP and IF assays. The results revealed that *p*-PYK2(Tyr402) could directly bind to STAT3, and the two proteins showed colocalization in the A549 and H460 cell lines (Fig. 5C, E). Co-IP of exogenously expressed PYK2 and STAT3 in HEK293T cells demonstrated the endogenous interaction of these two proteins (Fig. 5D). Nucleocytoplasmic separation and IF experiments in H1299 cells confirmed that PYK2 overexpression increased the nuclear accumulation of *p*-STAT3(Tyr705) (Fig. 5F-G).

**VGF is an important downstream target of the PYK2-STAT3 axis signaling**

**Fig. 6:**
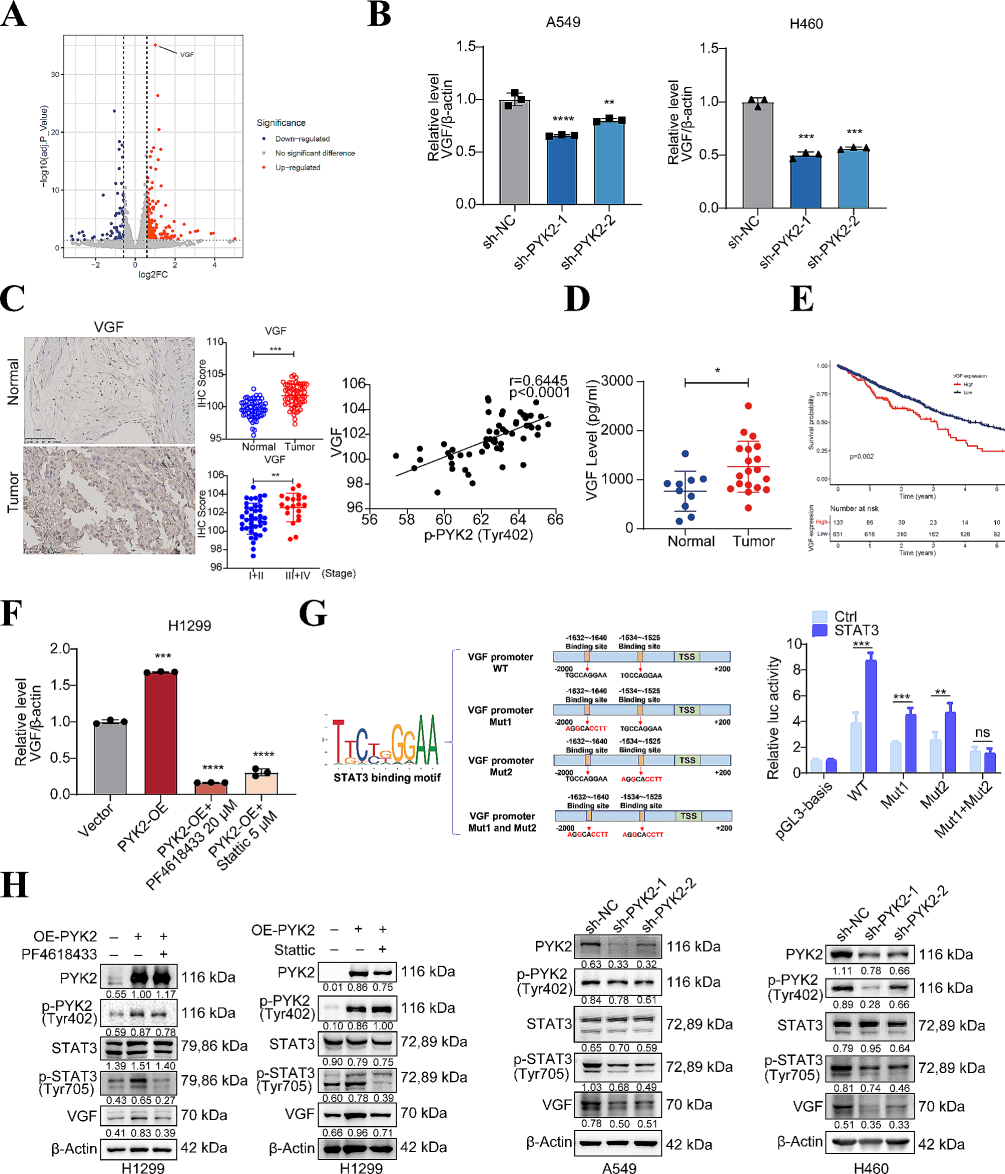
PYK2 regulates VGF expression through *p*-STAT3(Tyr705). **A** VGF in volcano plot of RNA sequencing based on H1299 PYK2 overexpressed cells compared to vector. **B** VGF mRNA reduced in two stable knockdown cell lines. **C** IHC of VGF; its scores were higher in tumor than paired adjacent normal lung tissue; higher in advanced TNM stage than earlier stage; IHC scores of *p*-PYK2(Tyr402) and VGF have a linear correlation. Scale bar, 100 μm. **D** Serum VGF of healthy control was lower than that in NSCLC patients. **E** Negative effect of VGF expression on overall survival prognosis of NSCLC patients from TCGA database. **F** Treatment with the *p*-PYK2(Tyr402) inhibitor PF4618433 20µMand *p*-STAT3(Tyr705) inhibitor Stattic 5µM reversed the increase in VGF mRNA. **G** The luciferase assay confirmed STAT3 transcriptionally regulated VGF. **H** Protein of VGF in stable knockdown cells and H1299 PYK2 OE cell lines with PF4618433 20µM and Stattic 5µM treated or not. * *P* < 0.05; ** *P* < 0.01; *** *P* < 0.001; **** *P* < 0.0001

To explore the mechanisms underlying the effects of PYK2, RNA sequencing data were analyzed using bioinformatics methods to obtain the transcriptional profiles of normal H1299 cells (vector) and H1299 cells stably overexpressing PYK2. A total of 284 differentially expressed genes were identified (false discovery rate < 0.05; log2FC > 0.585 or < -0.585), of which 207 were upregulated, and 77 were downregulated in PYK2-overexpressing cells. KEGG pathway enrichment for these genes(Fig S3A) and a volcano plot (Fig. 6A) confirmed the apparent correlation between VGF and the neuroactive ligand-receptor interaction pathway. The VGF mRNA and VGF protein levels in cell culture supernatant were consistent with PYK2 in three NSCLC stable cells (Fig. 6B, F and Figure S3B ). IHC of 57 paired tumor and normal tissues from lung cancer patients showed that the IHC scores of VGF were higher in tumor tissues than in normal tissue. Moreover, the scores were also significantly higher in advanced TNM stages than in early TNM stages. Especially, there was a positive linear correlation between the IHC scores of VGF and *p*-PYK2(Tyr402) (Pearson *r* = 0.6445, *P* < 0.0001) (Fig. 6C). VGF serum levels in 19 NSCLC patients were found higher secreted in NSCLC patients than in 10 healthy persons (Fig. 6D). Also VGF mRNA levels was found to have a negative impact on overall survival of NSCLC patients based on TCGA database(Fig. 6E).

**Knockdown VGF in H1299 vector and PYK2 overexpressing cells** **inhibit cell proliferation and migration, invasion.**

siRNA of VGF(listed in Table S4) was used in H1299 vector and PYK2 overexpressing cells, mRNA and protein were all knockdown (Figure S3C, F ).CCK-8 and EdU assay showed that, in H1299 vector cells, knockdown of VGF inhibit NSCLC proliferation *in vitro;* when knockdown VGF in PYK2 overexpressed cells, the enhanced growth of NSCLC cells were reversed(Figure S3 D-E). Similar results were found in transwell migration and Matrigel invasion assay (Figure S3 G). The protein level of Cyclin D1 was consistent with VGF protein too (Figure S3 F).

**PYK2 regulates VGF transcription through ***p*-STAT3(Tyr705).

**Fig. 7:**
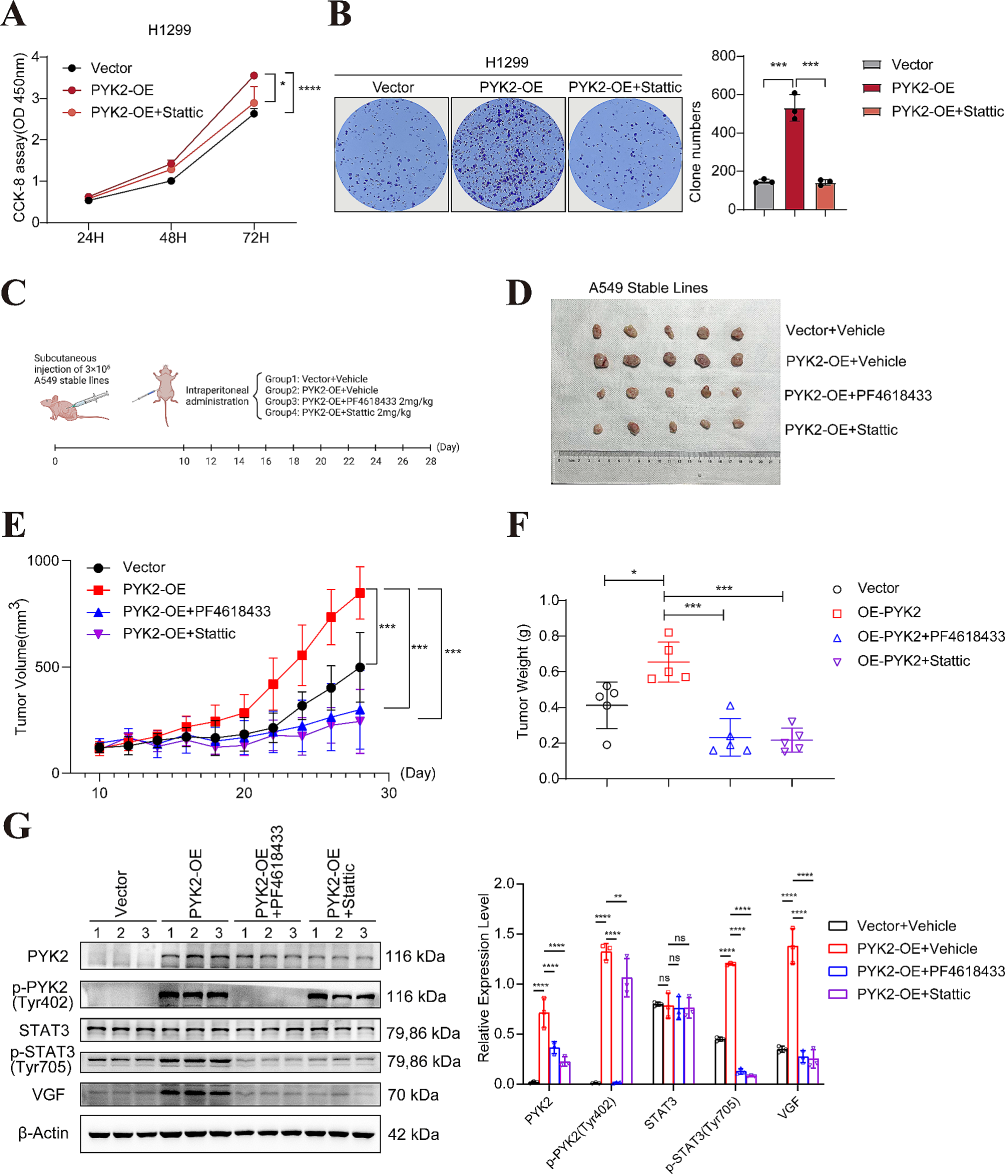
PYK2 inhibitor PF4618433 and STAT3 inhibitor Stattic reversed the enhancing effect of PYK2-overexpressing for xenograft subcutaneous tumor growth in vivo. **A-B** The rescue phenotypic experiments with Stattic 5µM attenuated the pro-cancer effect of PYK2 overexpression in CCK-8 and colony formation. **C** Flow chart of this animal experiment. **D-F** Tumor photos and growth curves, tumor weight at execution of vector, PYK2 OE, PYK2 OE + PF4618433, PYK2 OE + Stattic. **G** WB of each group for PYK2, *p*-PYK2(Tyr402), STAT3, *p*-STAT3(Tyr705) and VGF. * *P* < 0.05; ** *P* < 0.01; *** *P* < 0.001; **** *P* < 0.0001

*p*-STAT3(Tyr705) can act as a transcription factor [33]and translocate to the nucleus when PYK2 is overexpressed in our experiments(Fig. 5F-G). Hence, the effect of PYK2 on VGF expression may result from transcriptional regulation. The luciferase assay showed that overexpression of STAT3 increased the promoter activity of VGF while mutated binding sites resulted in diminished luciferase activity in A549 cells (Fig. 6G). In A549 and H460 PYK2 knockdown stable cells, VGF protein level reduced(Fig. 6H). In the H1299 PYK2 overexpressing cell line, treatment with the *p*-PYK2(Tyr402) inhibitor PF4618433 20µM(Fig.S4A-B) and *p*-STAT3(Tyr705) inhibitor Stattic 5µM(Fig.S4C-D) reversed the increase in VGF mRNA and protein expression (Fig. 6F, H). These findings indicate that PYK2 regulates VGF expression through *p*-STAT3(Tyr705). The rescue phenotypic experiments with Stattic 5µM attenuated the pro-cancer effect of PYK2 overexpression in CCK-8, colony formation, migration and invasion (Fig. 7A-B, Fig S1 C).

**PF4618433 and Stattic can reverse the enhanced growth of subcutaneous xenograft tumors ** in vivo.

We used two groups of A549 cells, one expressing an empty vector and the other stably overexpressing PYK2(Fig.S5A-F), to establish a tumor xenograft model in athymic BALB/c mice (Fig. 7C). The in vivo tumor growth curve and tumor mass data are presented in Fig. 7D-F. As shown in Fig. 7D-F, the PYK2 overexpression group had the highest growth rate, and both PF4618433 and Stattic (2 mg/kg) [27] inhibited the growth of these tumors. WB showed that total and active PYK2, STAT3, and VGF protein levels were higher in the PYK2 overexpression group than in the vector group. However, after inhibitor treatment, the protein levels of *p*-PYK2(Tyr 402), *p*-STAT3(Tyr 705), and VGF decreased (Fig. 7G). These in vivo results were consistent with those obtained in vitro. Overall, we confirmed that PYK2 promotes tumor growth in vivo, and a PYK2 inhibitor/STAT3 inhibitor can reverse this effect.

## Discussion

**Fig. 8:**
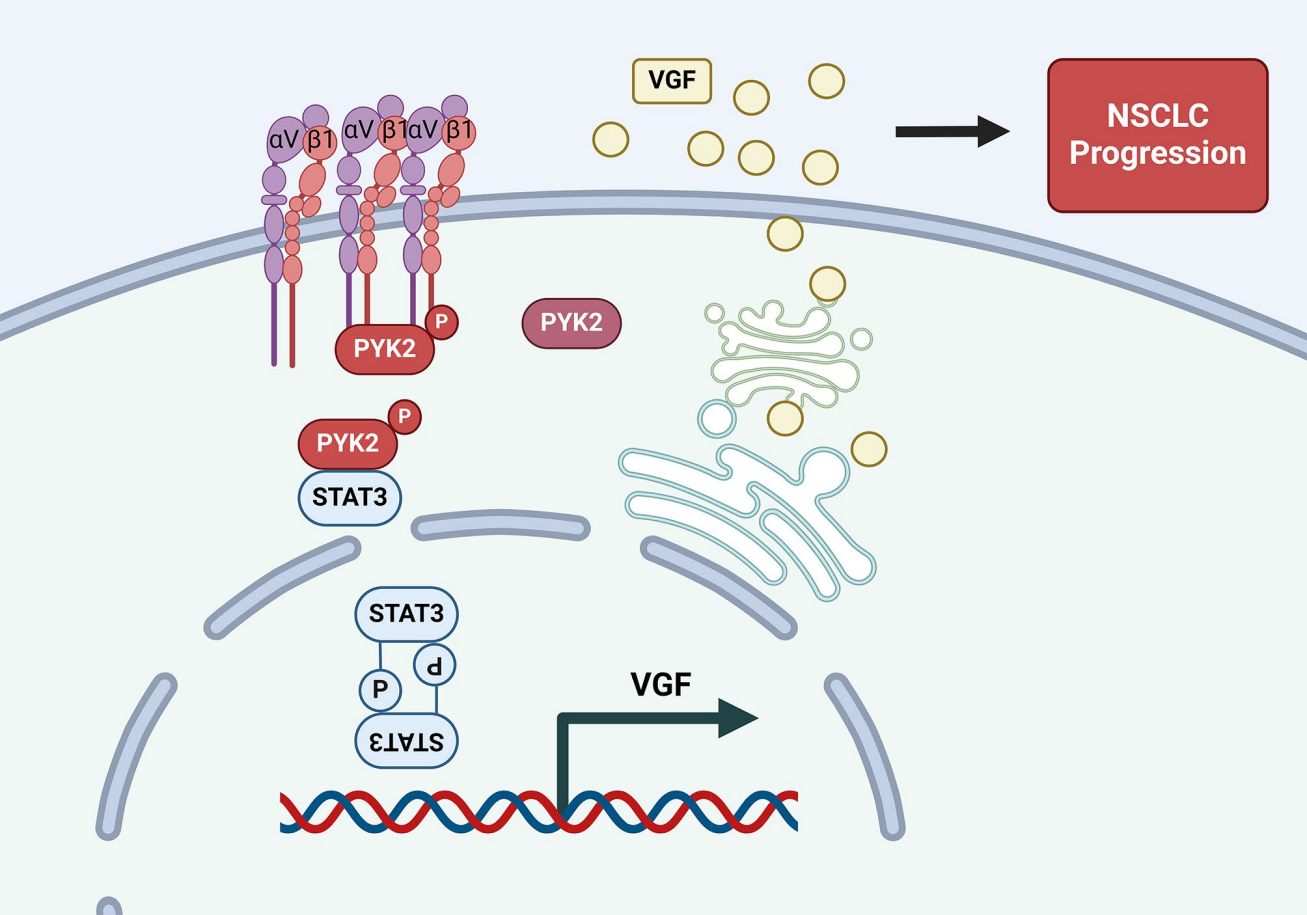
Pathway figure of this study

PYK2 is widely recognized as a tumor progression factor in various cancers [6–14, 24, 34], but its detailed mechanism in NSCLC is unknown. In this study, we found that PYK2 mRNA and protein levels were higher in NSCLC tumor tissues than in paired normal tissues, and high PYK2 expression was correlated with poor prognosis in NSCLC. This indicated that PYK2 play an integral role in the progression of NSCLC. In vitro experiments proved the tumor-promoting role of PYK2 and *p*-PYK2(Tyr402). Further, the findings demonstrated that integrins αV and β1 interact with PYK2 and regulate its phosphorylation at the Tyr 402 site. Subsequently, we also discovered that PYK2 interacts with the recognized oncogene and a protein kinase, STAT3. *p*-PYK2(Tyr402) then phosphorylates STAT3 to its activated form *p*-STAT3(Tyr705) and promotes the nuclear transport of *p*-STAT3(tyr705). Finally, these changes led to upregulated VGF mRNA and protein expression. Knockdown of VGF inhibited NSCLC progression and partially reversed cancer-promoting effect of overexpressing PYK2. Using animal experiments, we validated the anti-tumor effect of the chemical inhibitors PF4618433 and Stattic. The proposed pathway is summarized in Fig. 8.

Cell cycle dysregulation is a crucial feature of neoplastic transformation. High levels of cyclin D1 are linked to cancer development and progression [35]. In our study, PYK2 knockdown led to the decreased expression of cyclin D1 in NSCLC cell lines. This was accompanied by a reduction in the S and the G1 phase arrest in the A549 and H460 cell lines with PYK2 knockdown, indicating suppressed cell cycle progression. Interestingly, the opposite effect was observed in H1299 cells overexpressing PYK2. As reported in multiple myeloma [7], the relationship between PYK2 and cyclinD1 is consistent and could partially explain why PYK2 knockdown inhibits NSCLC cell proliferation and tumor growth.

Integrins comprise 18 alpha and eight beta subunits, giving rise to 24 distinct integrin heterodimers on the cell membrane [36]. Integrins bind to extracellular ligands and transmit signals to the cell’s interior. Integrins are closely linked to cancer progression due to their roles in cell proliferation, adhesion, and angiogenesis. Thus, integrins have been recognized as important targets for cancer therapy [37]. Recently, we demonstrated the relationship of integrins with tumor progression and drug resistance in NSCLC [25, 30, 32]. In this study, we identified the colocalization between PYK2 and integrin αVβ1 and found that integrin αVβ1 can regulate the phosphorylation of PYK2 at tyrosine 402 in NSCLC cells. Similar to the effects of integrins on FAK, only the active site such as *p*-FAK(Tyr397), *p*-PYK2 (Tyr402) are regulated while total FAK or PYK2 remain unchanged [25, 30]. This provides new insights into the relationship between the integrin family and the FAK family in the context of NSCLC.

The proline-rich sequences of PYK2 can bind to several Src homolog two and Src homolog3 (SH2, SH3) domain-containing proteins [6]. This is because the C-terminal region of STAT3 contains the SH2 domain [38], enabling their binding and interaction. Among the seven members of the STAT protein family, STAT3 is the most closely associated with tumor growth and immunosuppression [39]. STAT3 activity is abnormally elevated in over 70% of human cancers [40], and phosphorylated or activated STAT3 levels are associated with poor clinical outcomes in several solid and hematologic malignancies [33]. After the phosphorylation of Tyr 705, the STAT3 protein dimerizes and translocates to the nucleus, subsequently acting as a transcription factor [33]. Interestingly, in multiple myeloma, PYK2 has been found to enhance STAT3 phosphorylation in response to fibronectin and interleukin-6 co-stimulation [8]. PYK2 also enhances EGF-induced STAT3 phosphorylation in human breast cancer [41]. Our results showed the colocalization between PYK2 and STAT3 in the cytoplasm. We found that activated PYK2 can phosphorylate STAT3 at the Tyr 705 site in NSCLC for the first time. Immunoprecipitation experiments in parental cell lines and HEK293T cells demonstrated the direct physical interaction between these proteins, and IF assays confirmed their colocalization. Further, we observed a more significant nuclear fraction of *p*-STAT3(Tyr705) in PYK2-overexpressing cells. In mouse xenografts, treatment with the specific and selective small-molecule inhibitors of PYK2 and STAT3 (PF4618433 and Stattic, respectively [27, 42]) attenuated the enhanced tumor growth induced by PYK2 overexpression. PF4618433 is a new selective PYK2-specific inhibitor that shows inhibitory effects against tumor cells in vitro and in vivo. Thus, PF4618433 treatment could represent a potential therapeutic strategy for NSCLC with high levels of PYK2.

The VGF gene is known to be induced by neurotrophic factors, and it was initially found to be secreted by the nervous system and linked to neurological disorders. Today, several VGF-derived peptides have been identified [43]. VGF has also been linked to other diseases, including cancer. VGF enhances glucose-stimulated insulin secretion and can improve glucose tolerance in type 2 diabetes [44]. VGF also mediates cooperation between differentiated glioblastoma cells and stem-like tumor cells, enhancing tumor growth [45], and it promotes the development of pancreatic neuroendocrine neoplasms via PI3K/AKT/CREB signaling [46]. In lung cancer, VGF expression confers resistance to EGFR kinase inhibitors and activates the EMT pathway, and VGF knockdown renders H1299 cells more sensitive to cisplatin treatment [47, 48]. High VGF expression is associated with the advanced stages of small-cell lung cancer and is correlated with a poor prognosis in lung adenocarcinoma [48]. All in all, VGF acts as an enhancer of lung cancer progression. Our study used data from TCGA (https://portal.gdc.cancer.gov/) to find that VGF expression is related to a poor prognosis in NSCLC patients. We discovered that PYK2-STAT3 regulates VGF expression, and Stattic partially reverses the upregulation of VGF mRNA and protein levels induced by PYK2 overexpression in the H1299 cell line. The dual luciferase assay validated two sites at VGF promoter that can bind with STAT3. Also we found that knockdown of VGF inhibit cell proliferation and migration, invasion in NSCLC cells. The positive regulatory relationship between PYK2 and VGF further confirms the tumor-promoting role of PYK2 and highlights the potential of PYK2 and VGF as therapeutic targets.

Several studies have reported that PYK2 expression is correlated with cell aggregation, focal adhesion, interaction with the extracellular matrix, EMT, and tumor metastasis [15, 16, 24, 49–51]. Moreover, PYK2 affects tumor growth in vitro and in vivo [52, 53]. This is not paradoxical. Tumor cells receive growth signals from cell aggregation [12], and targeting multicellular tumor aggregation inhibits the growth of colon cancer [54]. PYK2 inhibition reduces cell growth more profoundly at lower cell densities in basal-like breast cancer cells [10]. Our study found that PYK2 can promote the proliferation, migration, and invasion of NSCLC cells and the growth of NSCLC xenografts in vivo. Integrin αVβ1 directly interacts with PYK2 and phosphorylates it in vitro, and activated PYK2 induces the phosphorylation and nuclear translocation of STAT3—this cascade increases VGF expression levels. PYK2 is an essential molecule in cancer progression. As a non-receptor tyrosine kinase [5], PYK2 occupies a central position in multiple signaling pathways. PYK2 responds to various signals, including growth factors, chemokines, cytokines, and integrins, and PYK2 is involved in multiple tumorigenic processes, including cell viability, growth, migration, invasion, EMT, and tumor angiogenesis [51].

The strength of this study lies in our detailed exploration of the relationship among integrins, PYK2, STAT3, and VGF in NSCLC. Moreover, we found that the selective inhibitors PF4618433 and Stattic could serve as therapeutic agents in some patients with NSCLC. However, one limitation was that this study was limited mainly to tumor cells. Hence, the interaction effects among normal cells, tumor cells, and the tumor microenvironment should be studied further.

### Electronic supplementary material

Below is the link to the electronic supplementary material.

Supplementary Material 1

Supplementary Material 2

Supplementary Material 3

Supplementary Material 4

Supplementary Material 5

Supplementary Material 6

Supplementary Material 7

Supplementary Material 8

Supplementary Material 9

Supplementary Material 10

Supplementary Material 11

Supplementary Material 12

Supplementary Material 13

Supplementary Material 14

Supplementary Material 15

## Data Availability

Data is provided within the manuscript or supplementary information files.
