# Supplementary figures and images for: Integrin αVβ1-activated PYK2 promotes the progression of non-small-cell lung cancer via the STAT3-VGF axis
Source: Cell Commun Signal. 2024 Jun 6;22:313. doi: 10.1186/s12964-024-01639-1 (PMC11157819; doi:10.1186/s12964-024-01639-1)

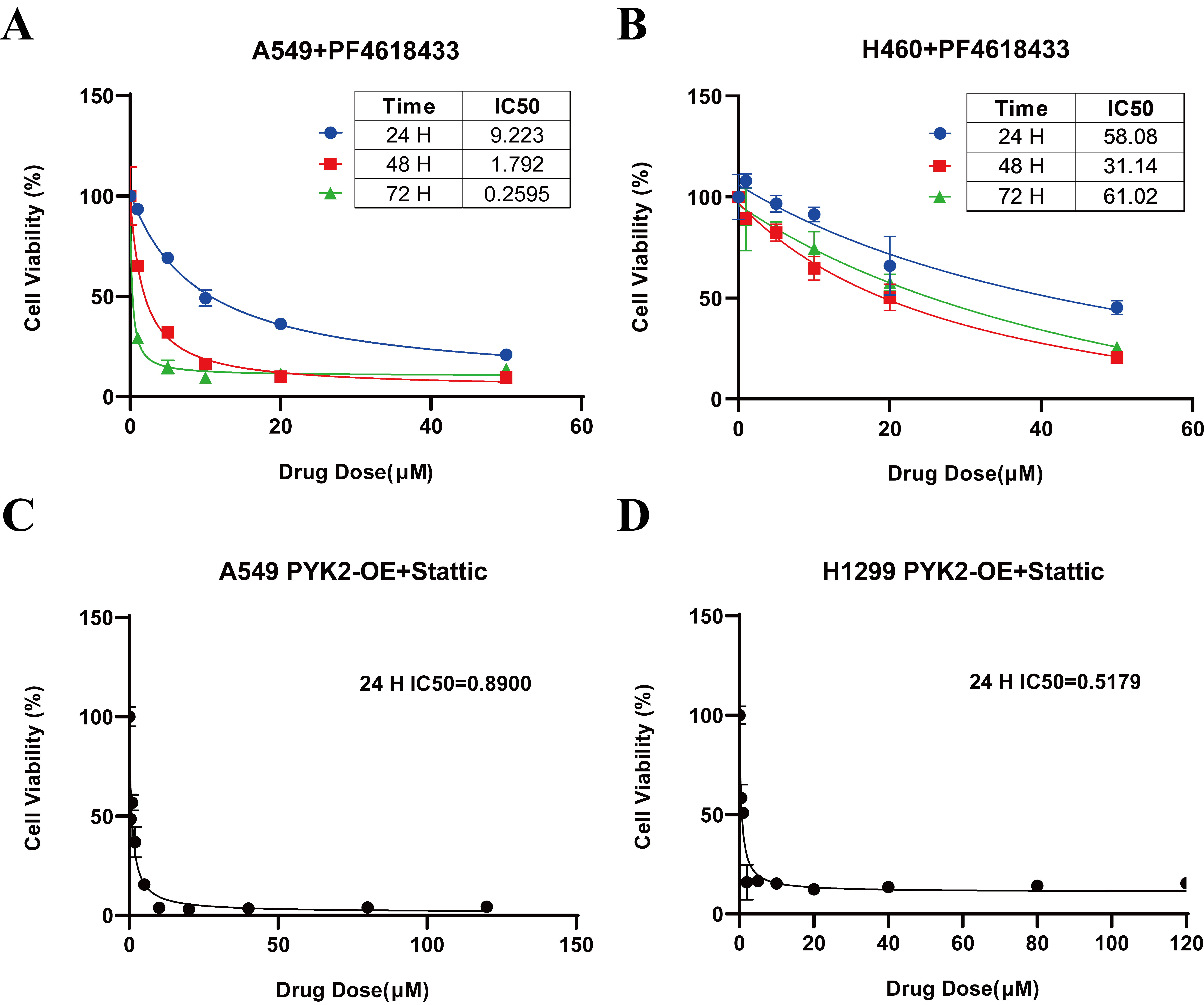

Supplement: Supplementary file 1 — Supplementary Material 1 [file 12964_2024_1639_MOESM1_ESM.png]

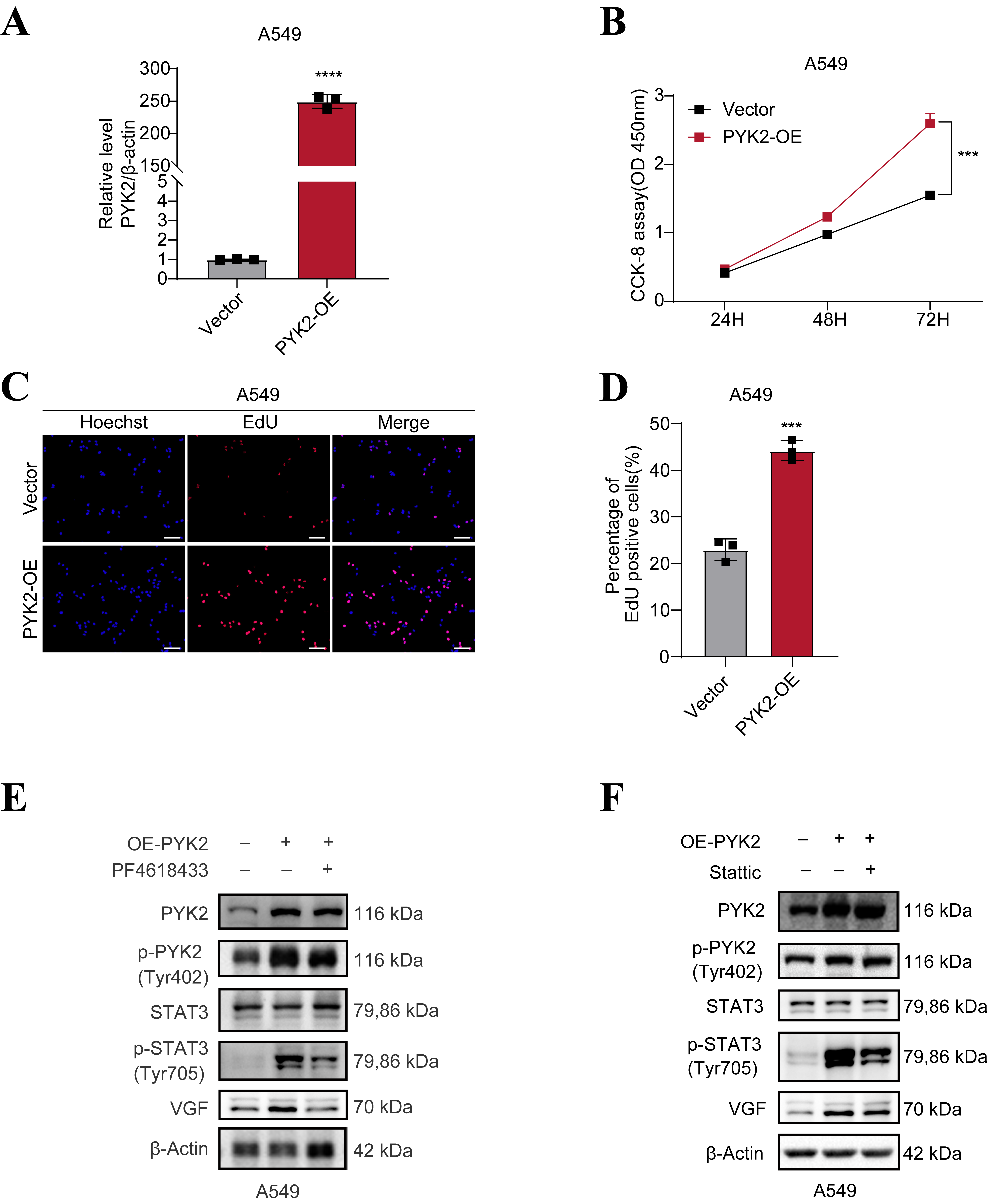

Supplement: Supplementary file 2 — Supplementary Material 2 [file 12964_2024_1639_MOESM2_ESM.png]

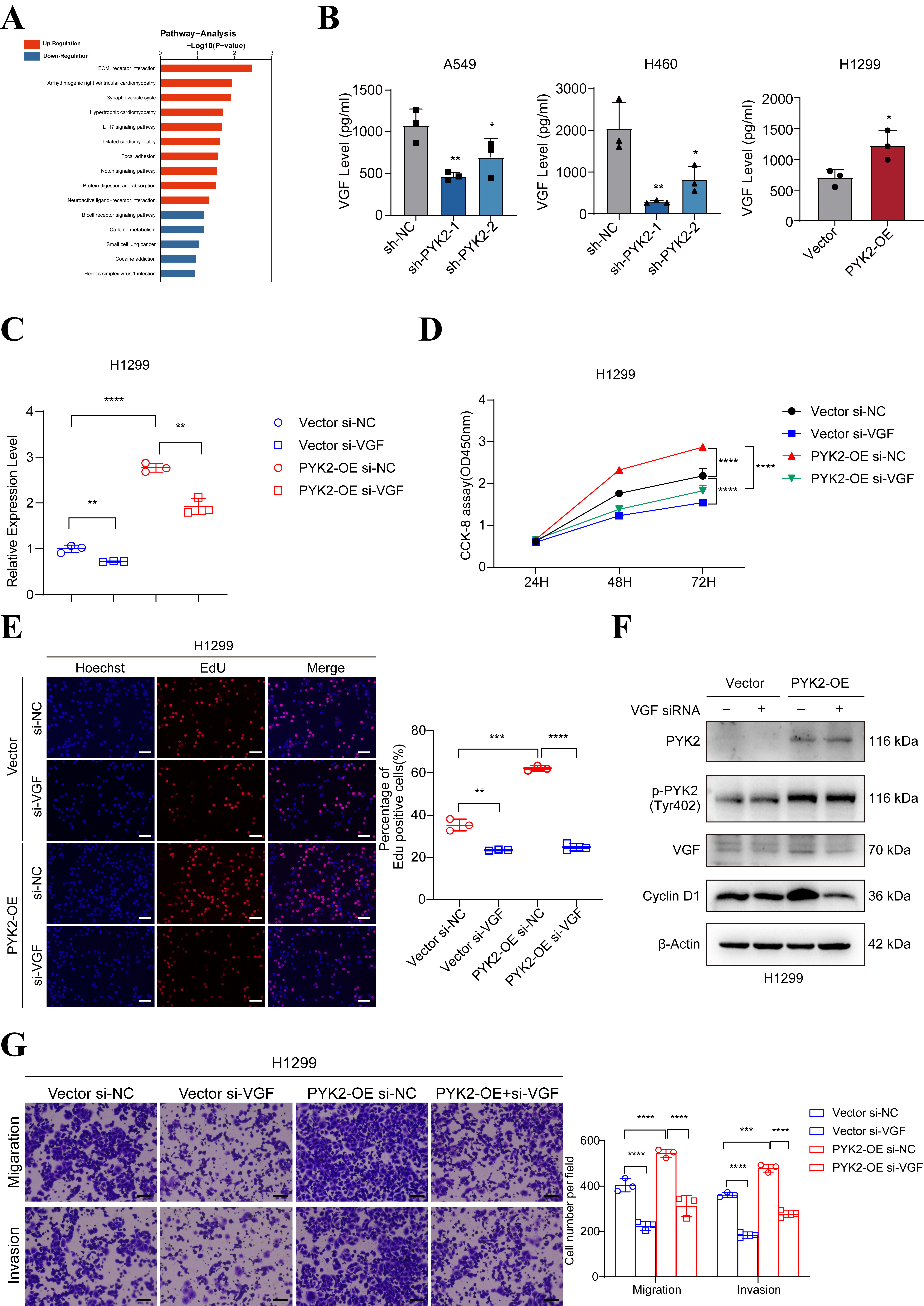

Supplement: Supplementary file 4 — Supplementary Material 4 [file 12964_2024_1639_MOESM4_ESM.png]

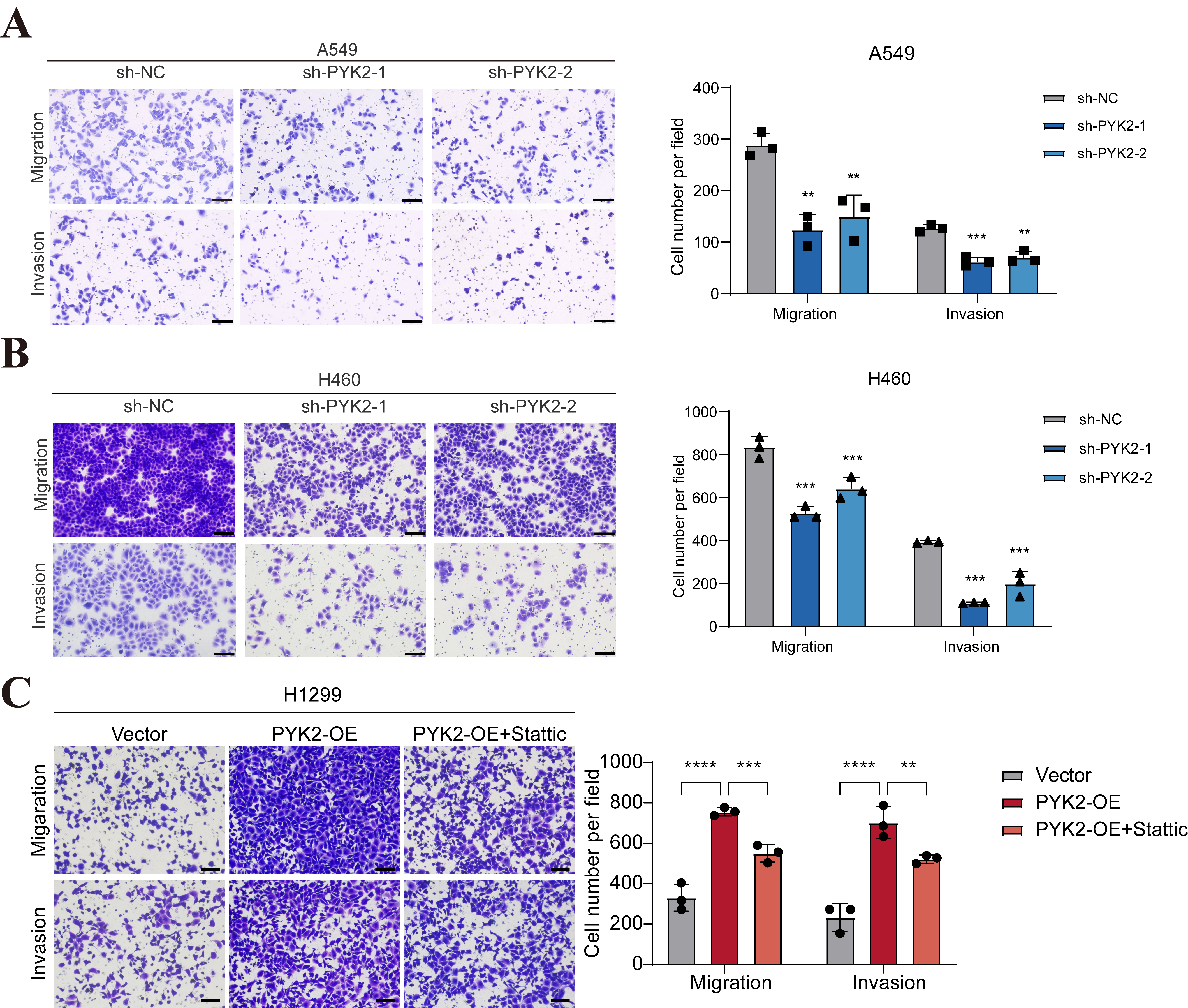

Supplement: Supplementary file 5 — Supplementary Material 5 [file 12964_2024_1639_MOESM5_ESM.png]

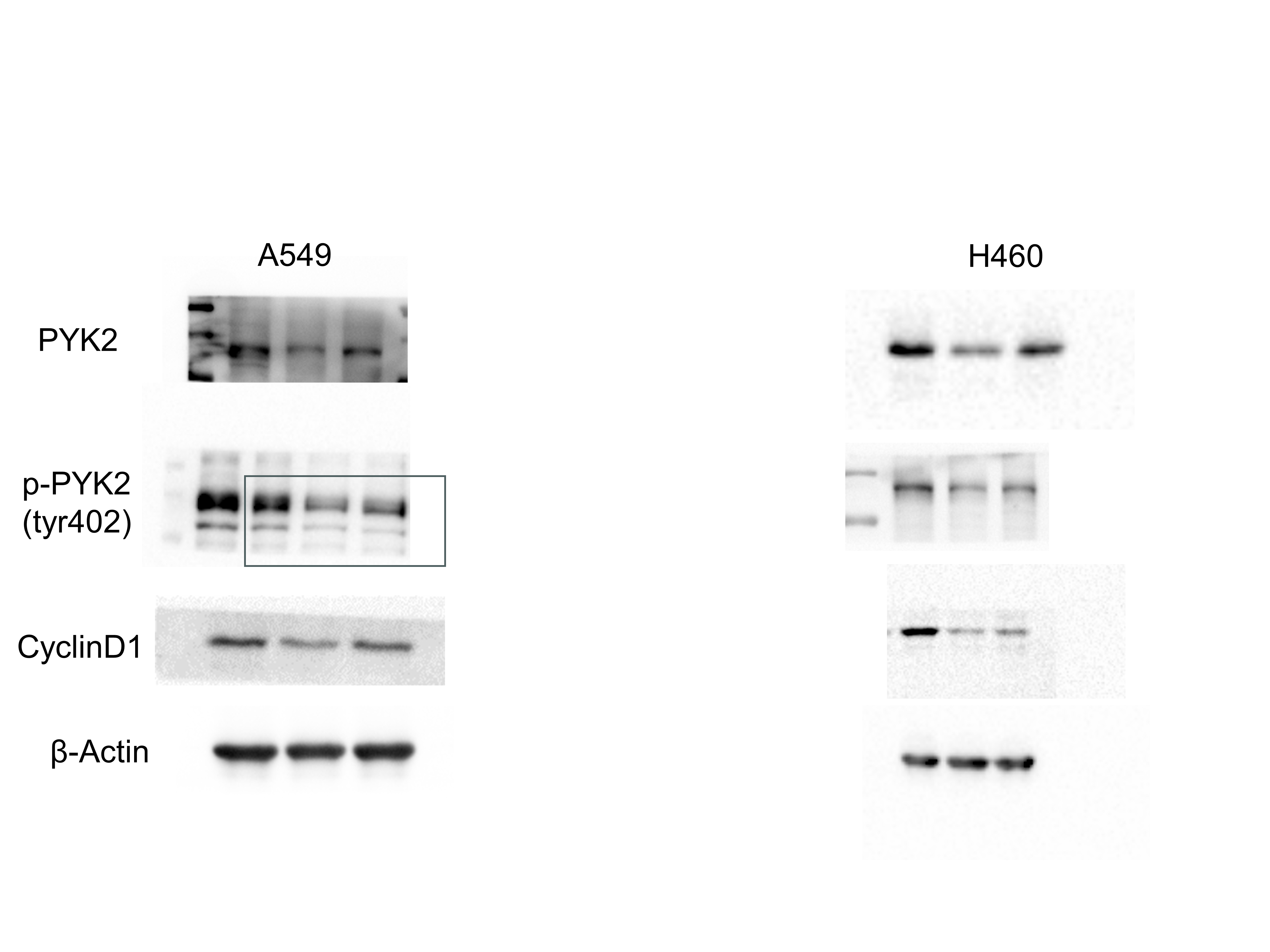

Supplement: Supplementary file 6 — Supplementary Material 6 [file 12964_2024_1639_MOESM6_ESM.png]

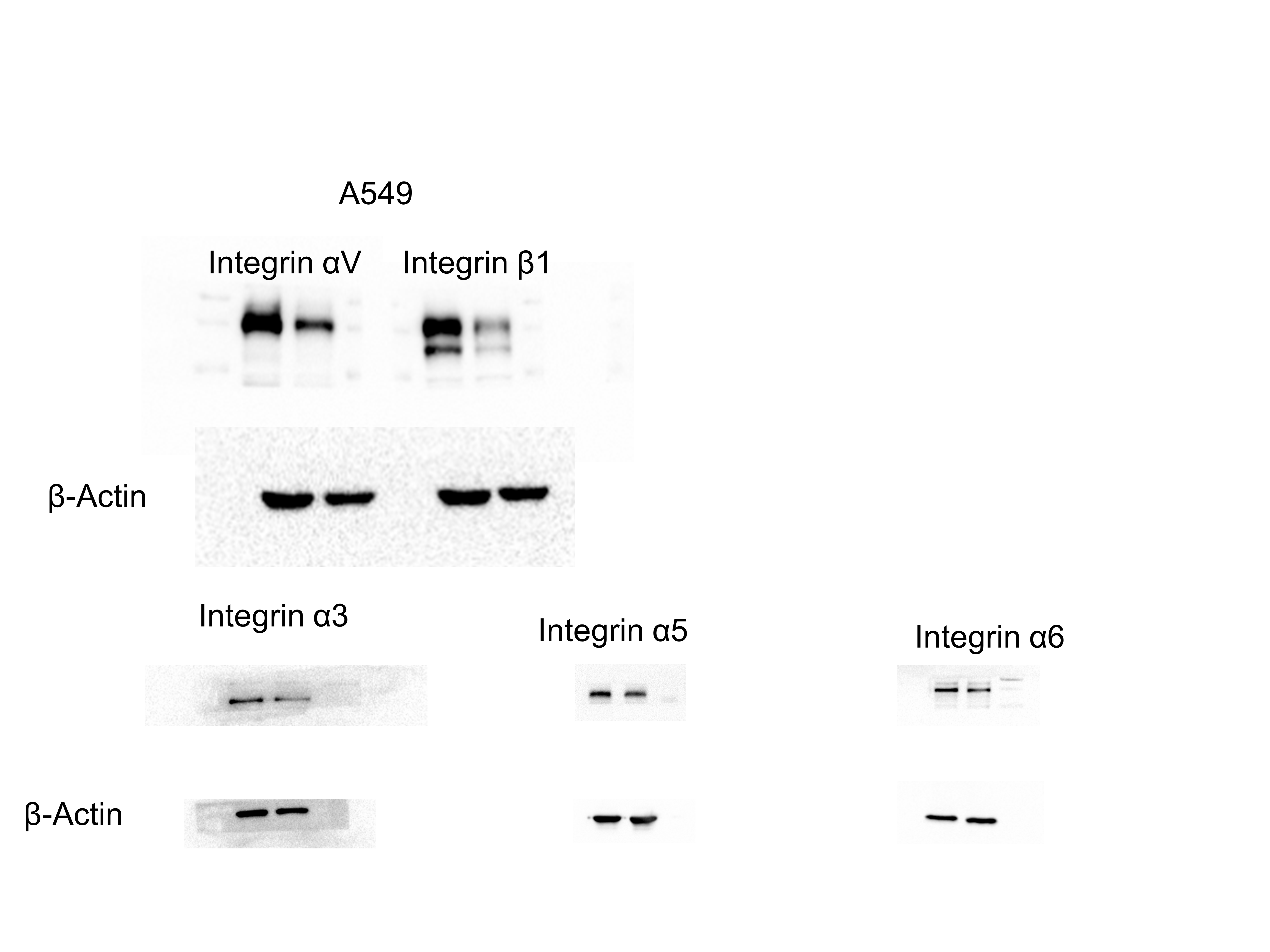

Supplement: Supplementary file 7 — Supplementary Material 7 [file 12964_2024_1639_MOESM7_ESM.png]

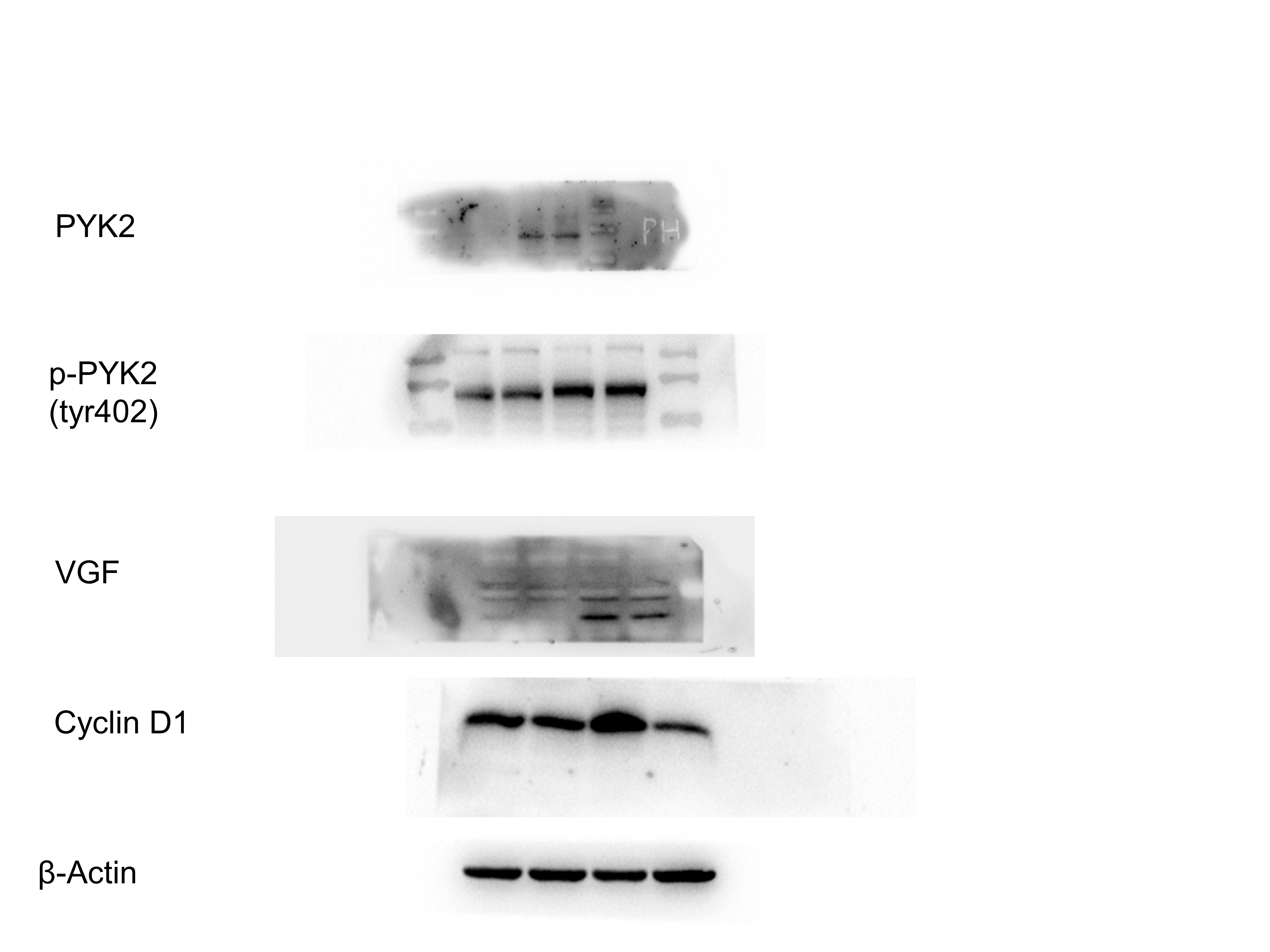

Supplement: Supplementary file 8 — Supplementary Material 8 [file 12964_2024_1639_MOESM8_ESM.png]

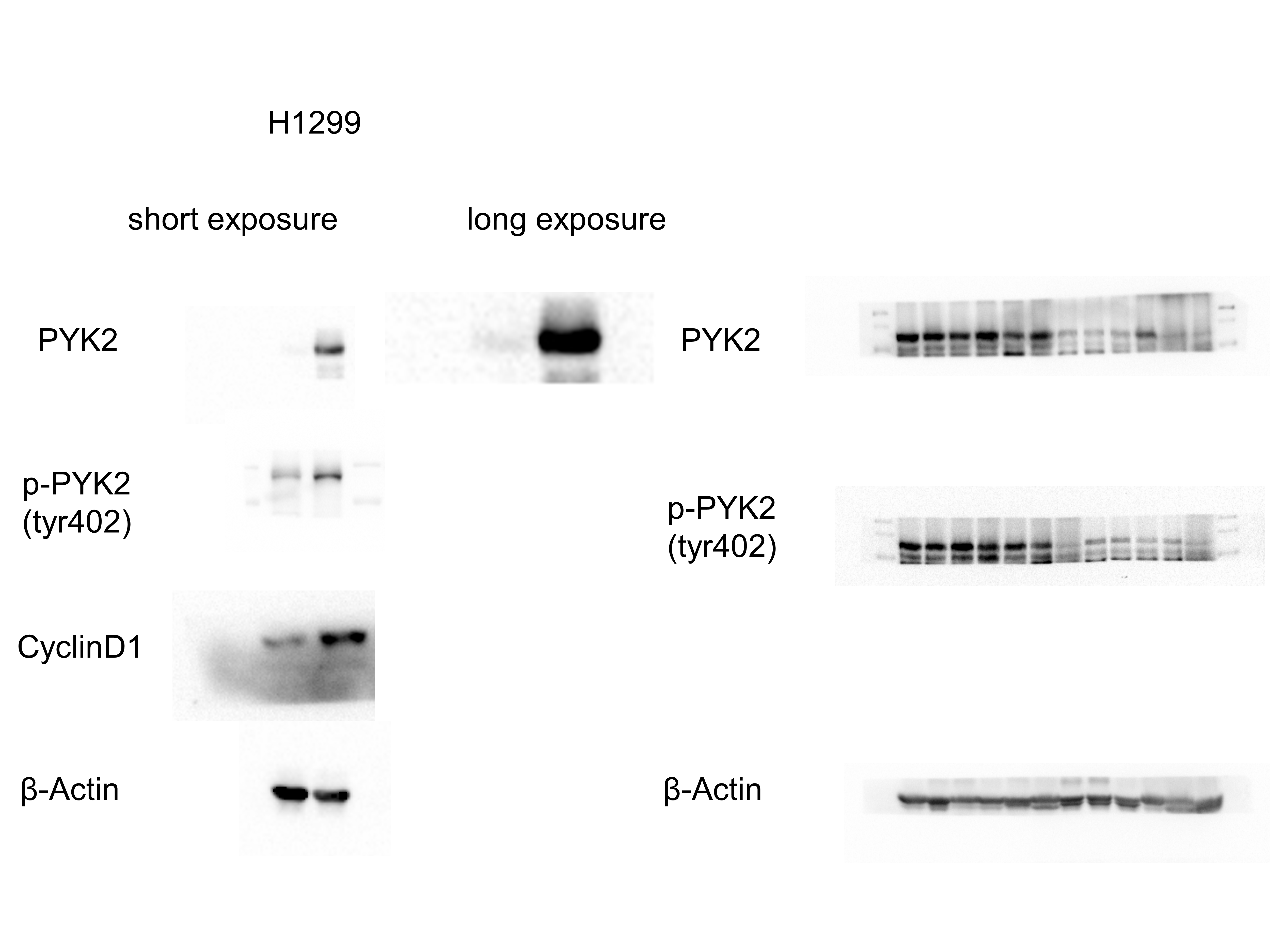

Supplement: Supplementary file 9 — Supplementary Material 9 [file 12964_2024_1639_MOESM9_ESM.png]

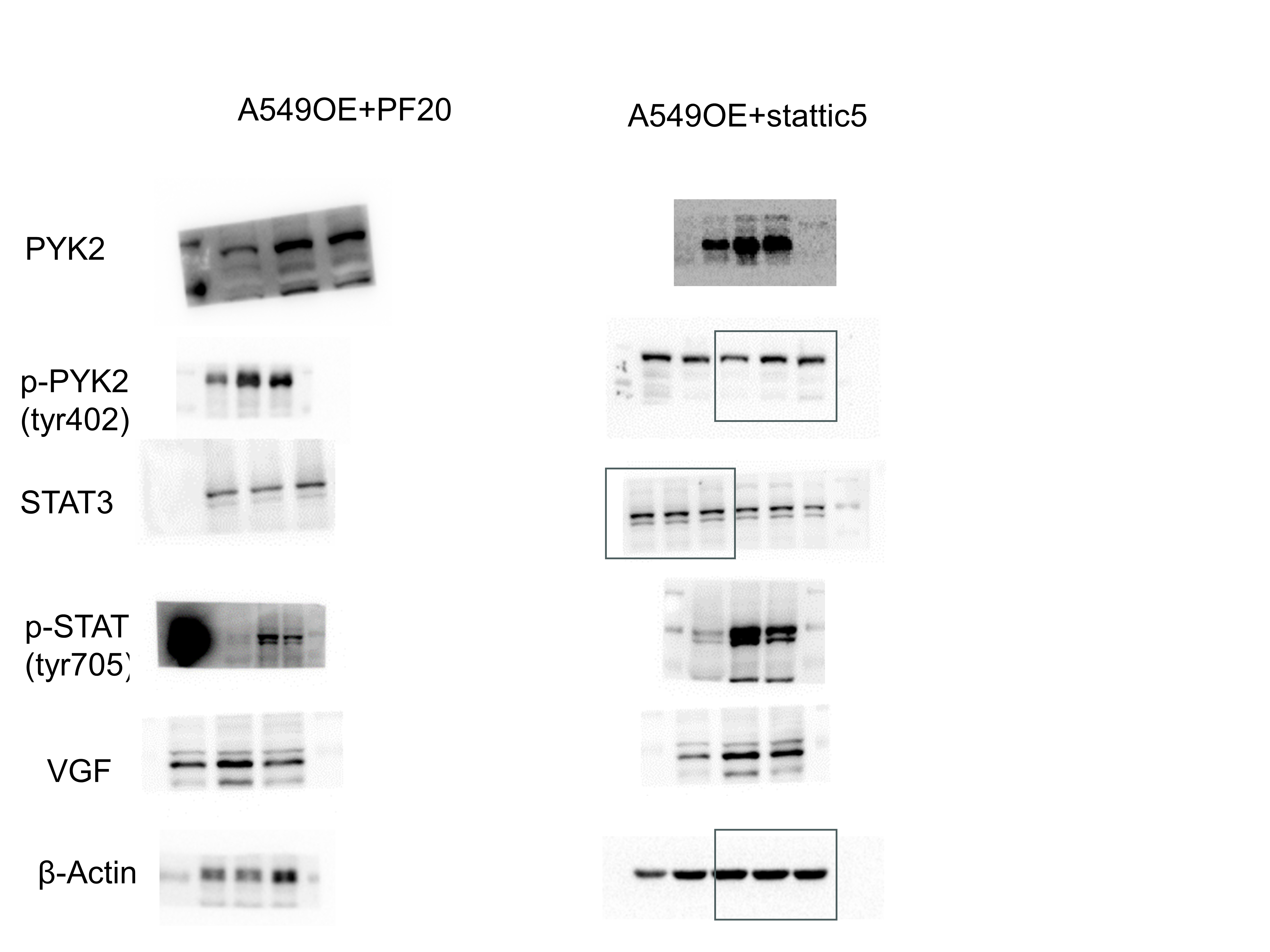

Supplement: Supplementary file 10 — Supplementary Material 10 [file 12964_2024_1639_MOESM10_ESM.png]

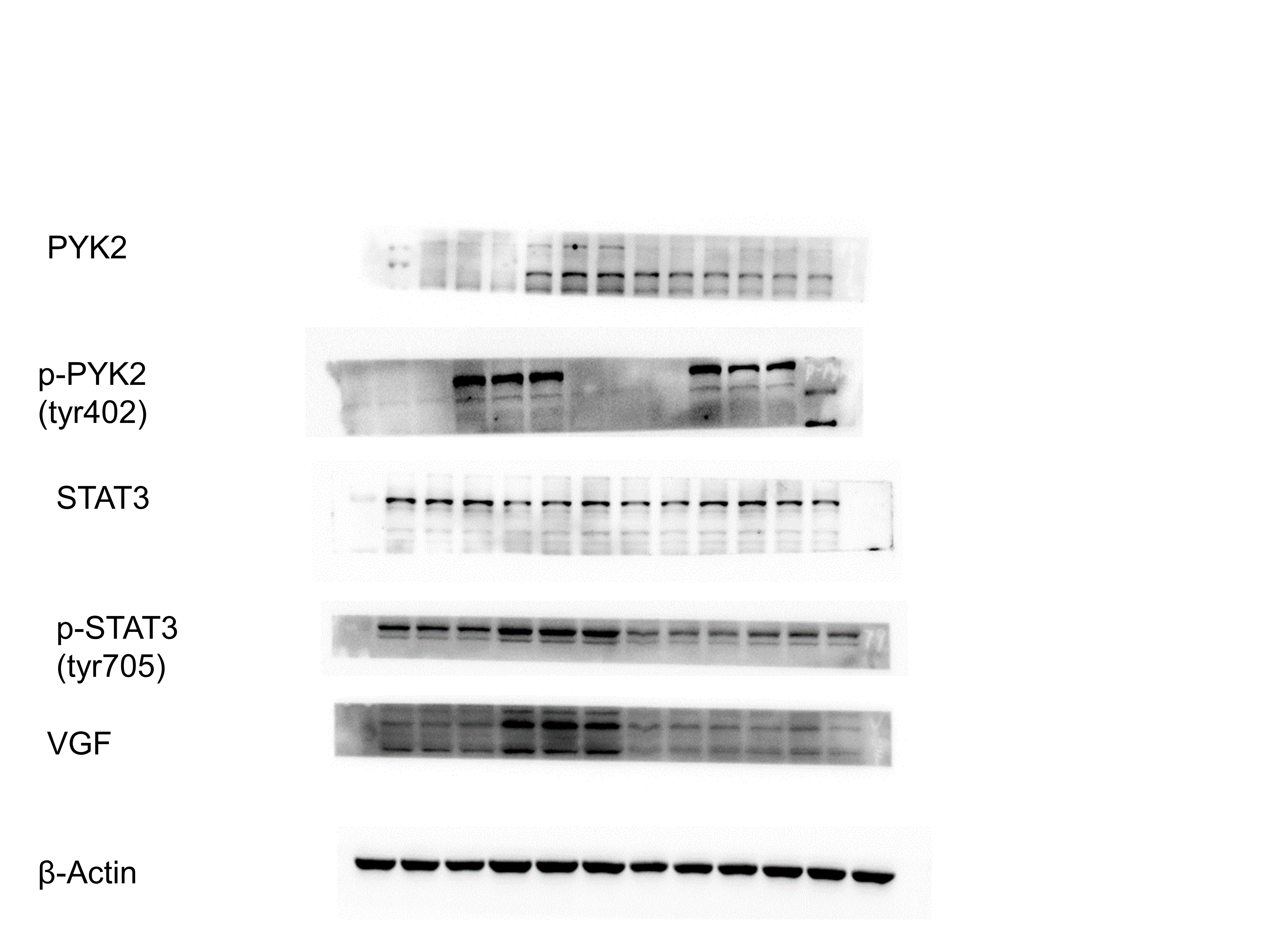

Supplement: Supplementary file 11 — Supplementary Material 11 [file 12964_2024_1639_MOESM11_ESM.png]

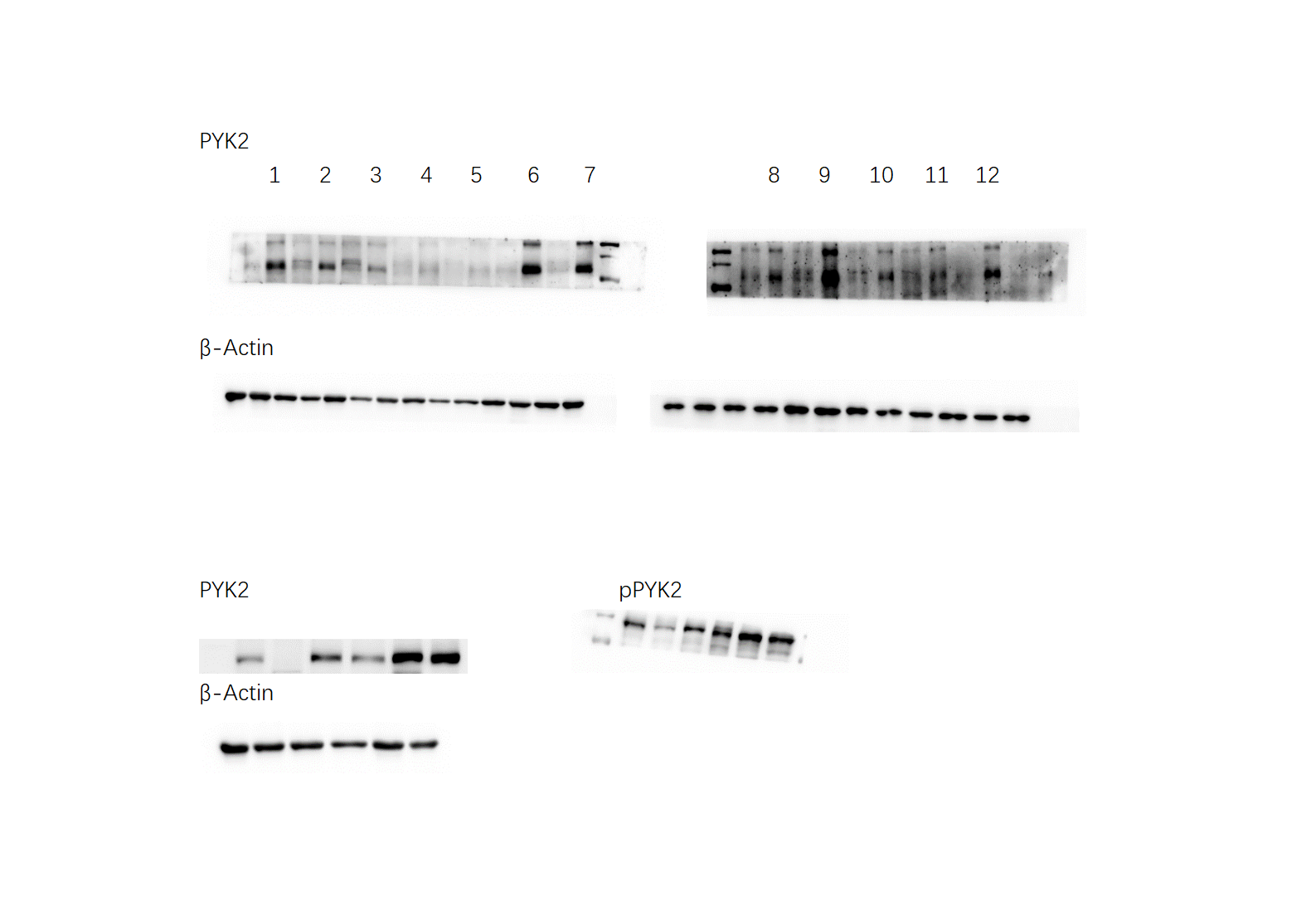

Supplement: Supplementary file 12 — Supplementary Material 12 [file 12964_2024_1639_MOESM12_ESM.png]

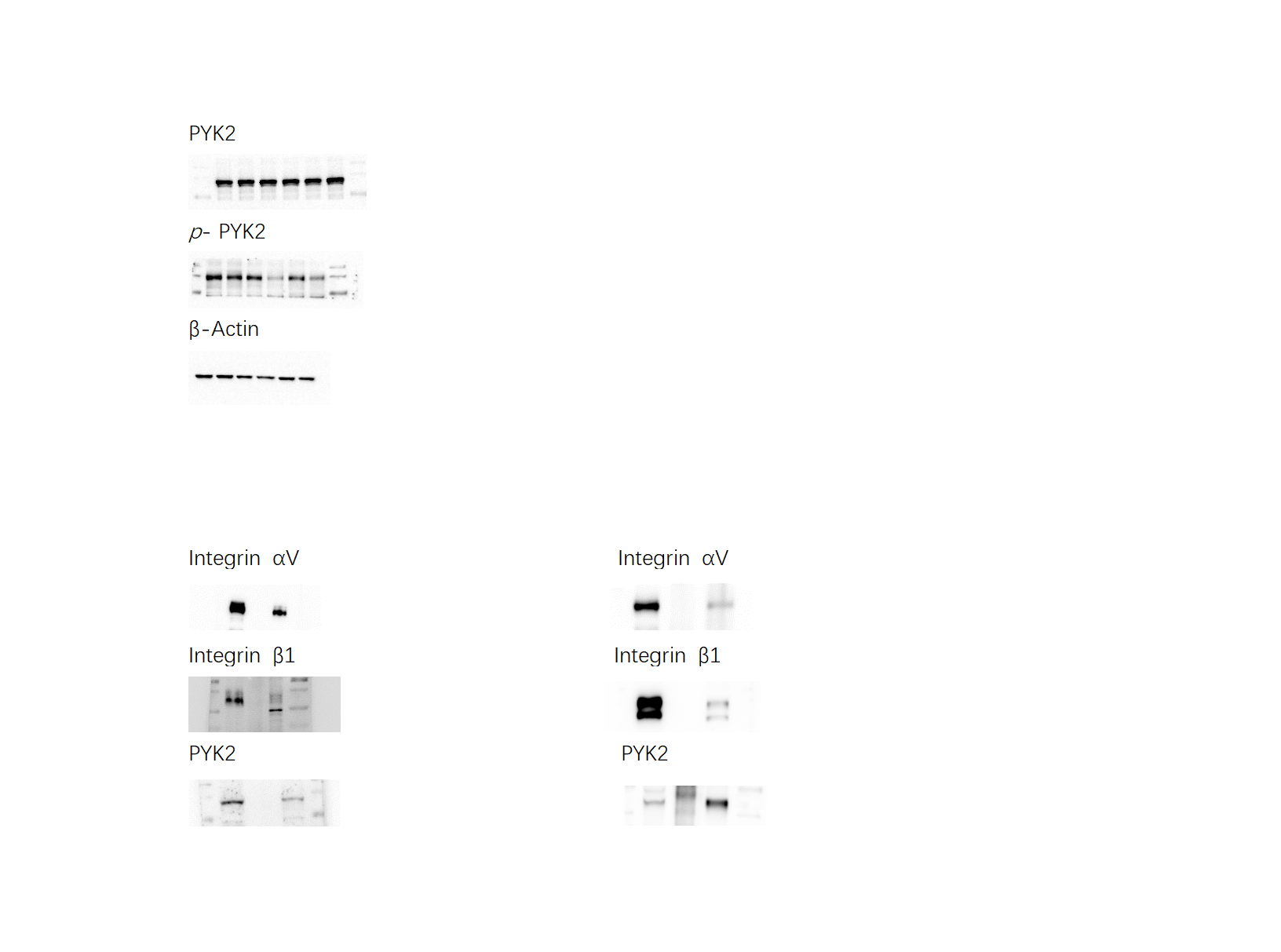

Supplement: Supplementary file 13 — Supplementary Material 13 [file 12964_2024_1639_MOESM13_ESM.png]

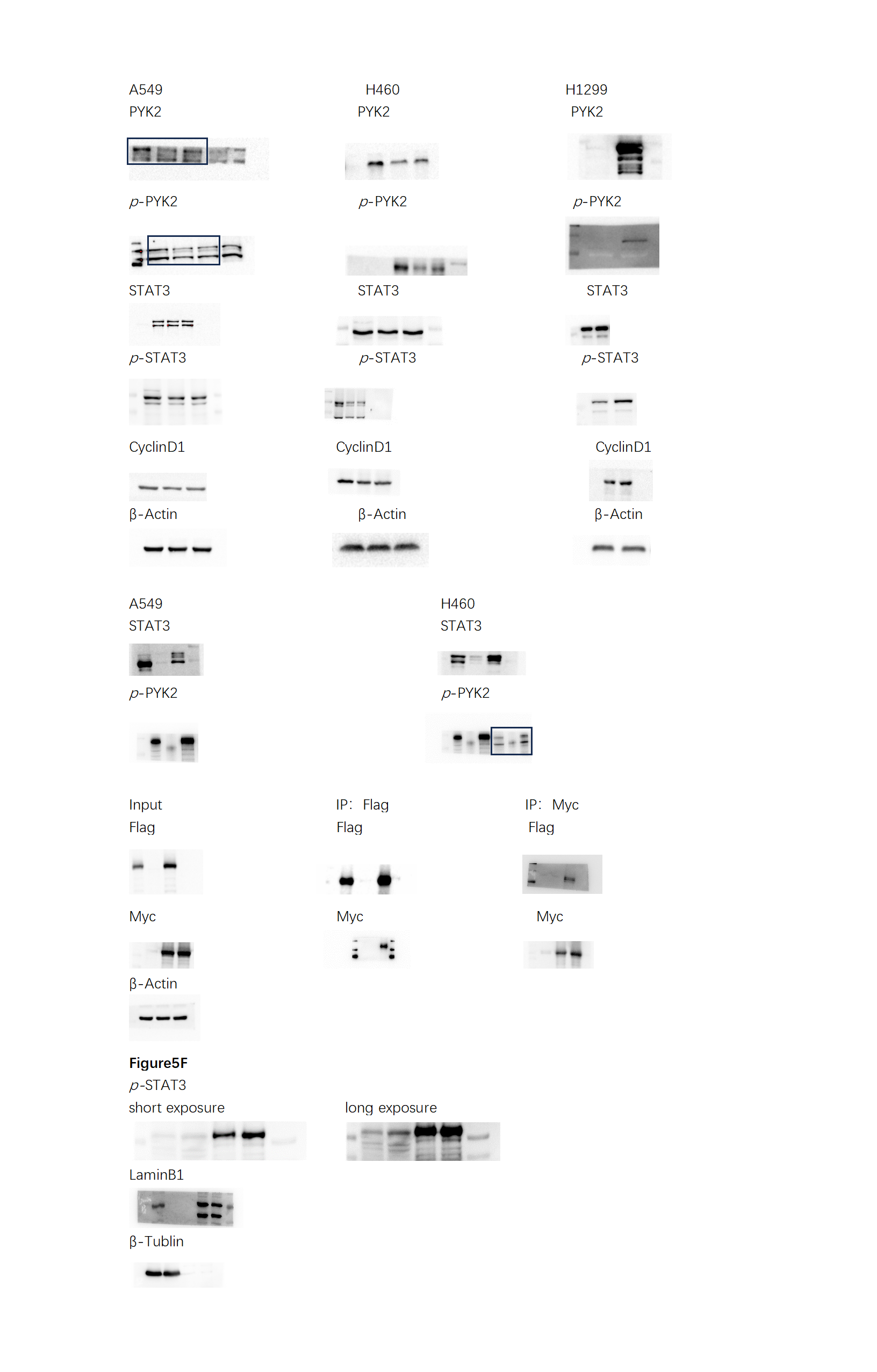

Supplement: Supplementary file 14 — Supplementary Material 14 [file 12964_2024_1639_MOESM14_ESM.png]

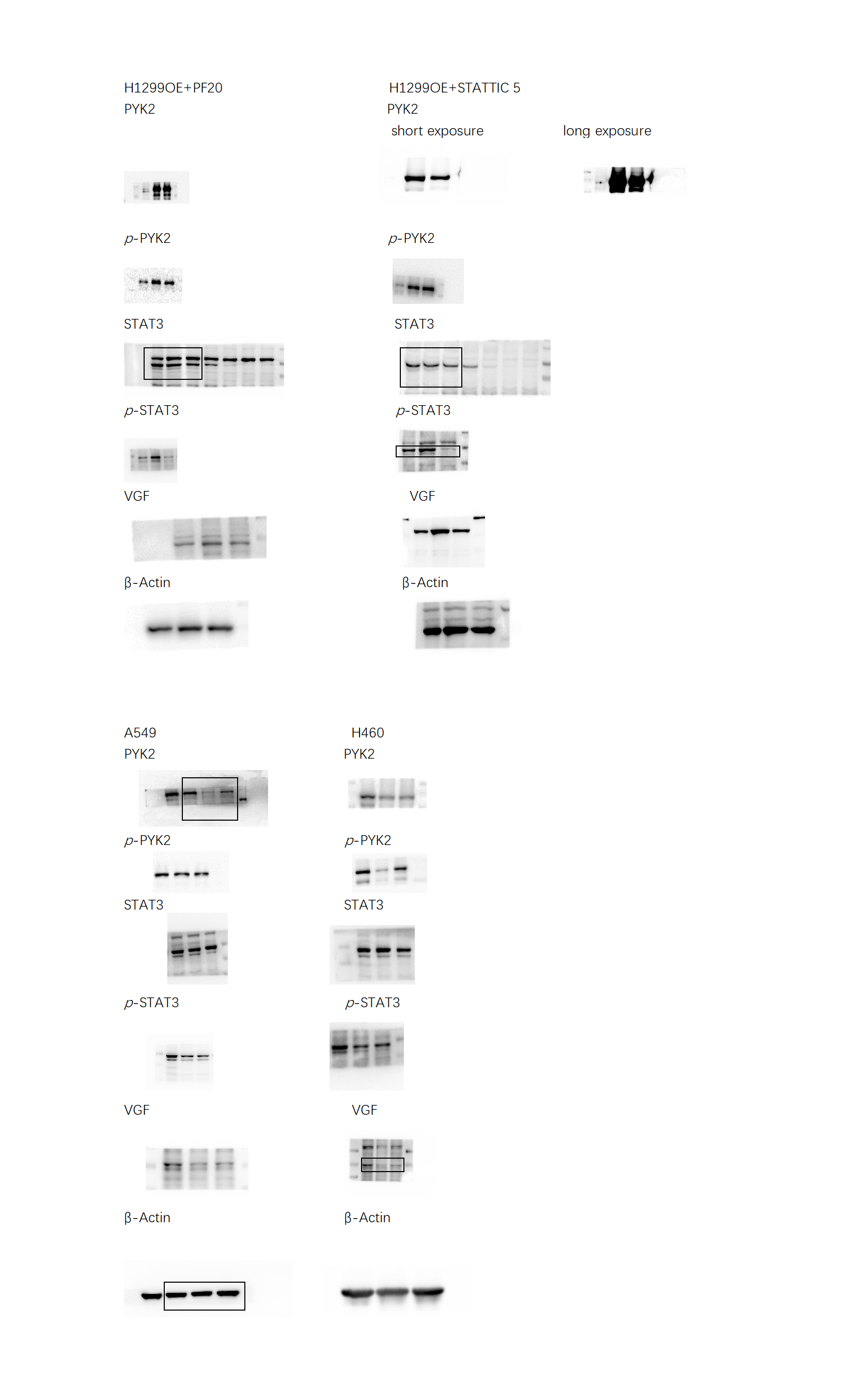

Supplement: Supplementary file 15 — Supplementary Material 15 [file 12964_2024_1639_MOESM15_ESM.png]
